# Supplementary material for: Designing human and generative AI collaboration: Trade-offs in productivity, satisfaction, and creative diversity
Source: PNAS Nexus. 2026 Nov 10;5(11):pgag353. doi: 10.1093/pnasnexus/pgag353 (PMC13749458; doi:10.1093/pnasnexus/pgag353)
Supplement: Supplementary file 1 [file pgag353_supplementary_data.pdf]

Supporting Information for

Designing Human and Generative AI Collaboration: Trade-offs in Productivity, Satisfaction, and Creative Diversity

Kartik Hosanagar and Daehwan Ahn

Corresponding Author: Kartik Hosanagar
Email: kartikh@wharton.upenn.edu

This PDF file includes:

Materials and Methods
Supplementary Text
Tables S0a-S0c and S1-S22
Figures S0-S2
SI References

A Methods

A.1 Study Design

Our experiment adhered to ethical guidelines and was approved by the University of Georgia's Human Research Protection Program (IRB ID: PROJECT00008677).

Participants. This study used data from a classroom exercise in an elective course on emerging technologies, where students applied prompt engineering principles to creative writing tasks. A total of 285 students successfully participated in the experiment.

Participant Recruitment. Students were invited to participate in a two-day study of AI support in creative writing. Each day included a 105-minute creative writing session and 15-minute surveys about the writing task and the participants' experience. Participants completed two 1,000-word creative writing tasks: one without AI support and one with AI support.

Participants received base course credit for participating. Additional credits were awarded for exceptional engagement and quality in responses to the reflection questions.

In accordance with ethical research guidelines and to uphold voluntary participation, students who chose not to participate in the study were given an alternative assignment with the same opportunity to earn bonus credits. This allowed students to choose whether to participate while preserving the research design.

We included all available story submissions from the experiment period in the analysis. While the recommended writing time was 105 minutes, a small number of participants (7 on Day 1 and 3 on Day 2) needed additional time to complete their stories. These observations remain in the analysis sample.

Experimental Design. Human-AI collaboration can be designed along several dimensions, including task allocation, decision-making authority, and user interface. We organize the designs around a process-based view from the creativity literature, which treats creative work as a sequence of pre-production and production phases rather than as a single activity (1, 2). In creative writing, pre-production includes ideation and outlining, and production includes drafting and editing. This process view yields four matrix designs (Table S13). The experiment focuses on three Day 2 human-AI collaboration designs: Human Creativity, Human Confirmation, and Copilot.

Given our emphasis on human-AI collaboration, Human Only serves as the Day 1 no-AI baseline rather than a focal Day 2 treatment. Similarly, a fully autonomous AI design, where AI performs all tasks without human oversight, is not a human-AI collaboration design. Instead, prior work has studied a design in which AI generates the idea, outline, draft, and edits while humans prompt, confirm, or reject the output (3, 4). We implement this condition as Human Confirmation.

Another design assigns humans responsibility for pre-production: generating ideas, selecting a concept, and outlining the story. AI then performs production tasks such as drafting and editing. We implement this condition as Human Creativity. A fourth possible design would have AI handle pre-production and humans manage production. We refer to this possible design as AI Creativity, but we did not study it. The remaining Day 2 design studied in the experiment is Copilot, in which participants could use AI assistance during both pre-production and production. This design resembles AI-assisted writing tools such as Microsoft Copilot and other AI-assisted systems.

The experiment evaluates three human-AI collaboration designs (detailed instructions for each group are provided in Section B.2):

- **Human Confirmation:** In this design, AI performs all tasks in both phases while humans prompt AI and confirm or reject AI output [e.g. Hitsuwari et al. (3)]. This design aims to "combine AI's advantages in low-cost idea [and content] production with the judgment of experienced humans to select the best option" (5). In our experiment, participants delegated the entire creative writing process—from ideation and outlining to drafting and editing—to ChatGPT. Their role was to write prompts and check the outputs.
- **Human Creativity:** This design uses task partitioning but keeps the pre-production tasks with humans. Participants generated ideas, selected a story concept, and outlined the story; ChatGPT then handled drafting and editing based on their prompts (6). Participants in our study independently developed story ideas and outlines before asking ChatGPT to draft and edit the narrative. During drafting and editing, their role was to prompt ChatGPT and check the outputs rather than directly write or edit the story themselves.
- **Copilot:** In this design, participants could use AI support at any stage of the creative process (7). In our implementation, participants worked with ChatGPT throughout ideation, outlining, drafting, and editing. They could start tasks independently and then ask for AI suggestions, or they could build iteratively on AI-generated suggestions.

Across all three designs, humans retain prompting and approval authority, but the allocation of creative control differs by condition. Human Confirmation leaves humans with final approval after AI generates the plan and draft. Human Creativity gives humans control over planning. Copilot lets writers use AI throughout planning and writing.

Day 1 Task: Writing without AI. On Day 1, participants completed an initial survey measuring their self-reported English fluency and creative writing abilities and experience (detailed questionnaires are presented in Section B.3.1). They also completed the Divergent Association Task (DAT), a psychological test measuring creativity that requires subjects to generate ten nouns as conceptually distinct from each other as possible. We selected the DAT because it is a brief, validated measure of verbal creativity, unlike more subjective and time-intensive creativity assessments (8). The DAT scoring methodology is detailed in Section B.3.2.

Following these preliminary assessments, participants were allotted 105 minutes to compose a 1,000-word creative story without AI assistance, establishing individual baselines for creative writing skill and ability. All participants received training in a seven-sequence story structure commonly employed in fiction writing (Section B.2.4). While encouraged to employ this structure for story outlining, participants with prior experience or alternative preferences were permitted to use different approaches. Upon completion, participants completed an exit survey documenting their time allocation across ideation, outlining, writing, and editing phases.

The survey also measured Process Satisfaction and three specific items: Flexibility, Process Effectiveness, and Intent to Reuse Process (Section B.3.3).

Day 2 Task: Writing with AI. On Day 2, participants were allotted 105 minutes to compose a new 1,000-word story with ChatGPT assistance. Before beginning the writing task, all participants received training in basic prompting strategies for the 105-minute task. The training covered few-shot prompting (providing examples of desired output), chain-of-thought reasoning (asking the model to reason step by step), emotional prompting (incorporating emotional elements), specificity (articulating clear creative objectives), and prompt experimentation.

Participants were randomly assigned to one of the three collaboration designs described earlier. While all groups had access to ChatGPT support, each was required to follow the assigned collaboration design (detailed instructions for each group are provided in Section B.2). Following task completion, participants completed an exit survey that incorporated all Day 1 survey elements plus additional measures of AI effectiveness (quality of suggestions and productivity improvement), satisfaction with the human-AI collaboration process, and perceived AI contribution to the final output (Section B.3.4).

Additional Task: Reflective Writing. Following completion of the two-day writing tasks and surveys, participants submitted two-page reflective essays addressing three areas: (1) their experience with AI collaboration in creative writing, including successful and unsuccessful aspects, (2) their recommendations for effective ways to collaborate with AI in writing, and (3) their strategies for prompting AI to achieve desired outcomes. We also collected all prompts and AI-generated outputs from the writing tasks. These qualitative materials—both the reflective essays and the prompt-output pairs—were analyzed to understand participants' experiences with AI-assisted writing.

Recruitment of Evaluators. In addition to participant evaluators, we recruited nine students from the same university as external evaluators. They were fluent in English and had taken writing courses or had other relevant experience. Each external evaluator was paid $15 per hour.

Evaluation. Story evaluation was conducted by two categories of assessors: internal (fellow participants) and external (research assistants), with all author identities and collaboration designs masked during evaluation. Each participant served as an internal evaluator for two stories.

Evaluators assessed each story on overall quality and four component ratings: originality, interestingness, writing quality, and coherence (Section B.4). Evaluators received only story texts, without information about authors or treatment conditions. The study's objectives were disclosed to external evaluators only after completion of all evaluations. Internal evaluators received explicit instructions to maintain objectivity, with emphasis on their assessments' importance for research validity and clarification that evaluations would not affect course grades.

Our experimental design combines a between-subject component, randomized assignment to one of three Day 2 collaboration designs, with a within-subject component, the Day 1 to Day 2 comparison for outcomes measured on both days. Figure S1 shows the experimental procedure; participant flow and balance checks are reported in Figure S0 and Tables S0a-S0c.

A.2 Data and Variables

Our two-day experiment enrolled and randomized 285 participants. Figure S0 and Table S0a report how participants and observations enter each analysis. The main sample for story quality analyses contains 549 participant-day story observations (278 from Day 1 and 271 from Day 2), with each story receiving four evaluator ratings (two internal and two external), yielding 2,196 evaluator-rating records. Each record includes one Overall Quality rating and four component ratings: Originality, Interestingness, Writing Quality, and Coherence.

Participant Characteristics. To account for individual differences, we collected data on four participant characteristics: self-reported writing experience, writing ability, English proficiency, and creativity. To measure creativity, we used 1) Day 1 performance as a skill indicator and 2) the Divergent Association Task (DAT), a validated instrument developed by Olson et al. (8) and used in prior work (9–11). The DAT quantifies divergent thinking in verbal creativity by having participants generate 10 maximally dissimilar nouns, then calculating the semantic distance between these words. Olson et al. reported a population average DAT Score of 78. Our participant cohort scored higher on average (mean DAT = 82.28; Figure S2).

Outcomes of Interest. We examine four primary outcomes in this study:

Total Completion Time: We use self-reported time spent on ideation, outlining, drafting, and editing to measure task efficiency across collaboration designs.

Story Quality: Following established literature (12–15), we assess overall story quality and component ratings. Human evaluators rate stories on a 7-point Likert scale across overall quality, originality, interestingness, writing quality, and coherence. The evaluation protocol and questionnaires are detailed in Section B.4.

User Satisfaction: We assess participant satisfaction through exit surveys administered on both days. The surveys measure overall process satisfaction and three specific items: Flexibility, Process Effectiveness, and Intent to Reuse Process (Section B.3.3). Day 2 surveys include additional measures specific to AI assistance: effectiveness ratings, satisfaction with AI collaboration, and perceived AI contribution to the final output (Section B.3.4).

Content Diversity: We examine AI's influence on story characteristics through genre distribution and genre counts, genre-label similarity within each condition, and semantic similarity among stories.

Definitions of Variables. Detailed survey questionnaires covering these variables are provided in Sections B.2 and B.3.

A.2.1 Participant Characteristics

- **DAT Score:** This measures participants' verbal creativity using the Divergent Association Task (DAT), in which participants list ten nouns that are as different from each other as possible.

- **Creative Writing Experience:** Self-assessed level of experience in creative writing, categorized as: (1) Limited exposure, (2) No professional experience but some experience, or (3) Some professional or academic experience.
- **Creative Writing Ability:** Self-assessed creative writing ability compared to peers, categorized as: (1) Below average, (2) Average, or (3) Above average.
- **English Language Proficiency:** Self-assessed comfort level with the English language, categorized as: (1) Not fluent, (2) Not-native but fluent, or (3) Native communicator.

A.2.2 Quality Metrics

- **Overall Quality:** The effectiveness of the story in conveying its main ideas creatively, interestingly, coherently, and with good writing, measured on a 7-point Likert scale from "Extremely bad" to "Extremely good".
- **Originality**: The originality of the story, including novel themes, plot, setting, structure, ending, and style, assessed on a 7-point Likert scale from "Extremely unoriginal" to "Extremely original".
- **Interestingness:** The story's ability to provide an engaging and immersive experience for readers through literary devices and vivid imagery, evaluated on a 7-point Likert scale from "Extremely uninteresting" to "Extremely interesting".
- **Writing Quality**: The clarity and comprehensibility of the language, including correct grammar, punctuation, and spelling, rated on a 7-point Likert scale from "Extremely poor writing quality" to "Extremely good writing quality".
- **Coherence:** The level of organization and structure in the story, judged on a 7-point Likert scale from "Extremely poor coherence" to "Extremely good coherence".

A.2.3 Self-reported Satisfaction with the Writing Process

- **Process Satisfaction:** Overall satisfaction level with the writing process, measured on a 7-point Likert scale from "Extremely dissatisfied" to "Extremely satisfied".
- **Flexibility:** The degree to which the writing process allowed for flexibility in achieving desired outcomes, assessed on a 7-point Likert scale from "Extremely disagree" to "Extremely agree".
- **Process Effectiveness:** Confidence in achieving writing goals using this process, rated on a 7-point Likert scale from "Extremely disagree" to "Extremely agree".
- **Intent to Reuse Process:** Likelihood of using the same writing process in the future based on its effectiveness, indicated as a binary Yes/No response.

A.2.4 AI Collaboration Evaluation (Day 2 Only)

- **Satisfaction with AI:** Overall satisfaction with the human-AI collaboration experience, evaluated on a 7-point Likert scale from "Extremely dissatisfied" to "Extremely satisfied".
- **AI Effectiveness:** Satisfaction with AI's assistance in writing, focusing on suggestion quality and perceived productivity, measured on a 7-point Likert scale from "Extremely dissatisfied" to "Extremely satisfied".
- **AI's Contribution:** Percentage of ChatGPT's contribution to story generation, specified on a scale from 0% (user contributed 100%) to 100% (ChatGPT contributed 100%).

A.3 Analysis Details

Regression Specifications. We use three sets of analyses: models for differences between conditions on Day 2 with participant characteristics included as controls, two-wave models with participant fixed effects for changes from Day 1 to Day 2 and contrasts across collaboration designs, and paired within-person tests for changes within each collaboration design. Participant fixed effects absorb time-invariant participant characteristics and therefore do not separately estimate DAT Score, Creative Writing Experience, Creative Writing Ability, or English Language Proficiency. Standard errors in the fixed-effects models are clustered by participant.

Randomization and Balance Checks. Participants were randomly assigned to one of the three Day 2 collaboration designs: Human Creativity, Human Confirmation, and Copilot. We assessed baseline balance in two complementary ways, using the samples summarized in Table S0a.

The first check covers the four participant characteristics measured before the Day 2 writing task with AI assistance: DAT Score, Creative Writing Experience, Creative Writing Ability, and English Language Proficiency ($N = 280$). The second check adds ten Day 1 baseline outcomes and uses the same 256 participants for all fourteen baseline variables. These outcomes are the five Day 1 quality ratings (Overall Quality, Originality, Interestingness, Writing Quality, and Coherence), the four Day 1 process satisfaction measures (Process Satisfaction, Flexibility, Process Effectiveness, and Intent to Reuse Process), and Day 1 Total Completion Time.

For each baseline variable, we tested equality of means across the three groups with an omnibus analysis of variance (ANOVA). Table S0b reports each test twice: once using all participants who have the variable in that row, and once using the same 256 participants who have all fourteen baseline variables. It also reports unadjusted pairwise Welch tests descriptively. No omnibus test survives a Bonferroni correction for the fourteen variables ($\alpha = .05/14 = .00357$).

Joint balance was tested with likelihood ratio tests from multinomial logistic regression models that predict randomized group assignment from the baseline variables (Table S0c). Neither joint test is statistically significant. The four participant characteristics give $\chi^2(8) = 12.67$, $p = .124$ ($N = 280$), and the full fourteen-variable baseline set gives $\chi^2(28) = 28.97$, $p = .414$ ($N = 256$).

The two checks answer different questions. The first asks whether the randomized groups were balanced on the measured participant characteristics before the Day 2 treatment. The second asks whether they were also balanced on the Day 1 baseline outcomes that matter for interpreting changes from Day 1 to Day 2.

Day 2 comparison models with participant characteristics included as controls. For outcomes measured at the participant level on Day 2, including the process satisfaction measures and AI assistance ratings in Tables S11 and S12, we estimate

$$y_i = \beta_0 + \beta_1 \text{Creativity}_i + \beta_2 \text{Copilot}_i + \theta_{\text{char}(i)} + e_i,$$

where Human Confirmation is the omitted reference group, Creativity_i indicates Human Creativity, Copilot_i indicates Copilot, and $\theta_{\text{char}(i)}$ denotes a vector of observed participant characteristics (DAT Score, Creative Writing Experience, Creative Writing Ability, and English Language Proficiency), which are included as controls and are not participant fixed effects. For quality outcomes measured at the rating level on Day 2 in Tables S4 and S7, the corresponding evaluator-level specification is

$$y_{ir} = \beta_0 + \beta_1 \text{Creativity}_i + \beta_2 \text{Copilot}_i + \theta_{\text{char}(i)} + e_{ir},$$

where r indexes evaluator ratings of participant i's story.

Inequality results. For the inequality analyses in Tables S6-S9, each panel estimates the association between the outcome on Day 2 and the corresponding measure from Day 1 within a collaboration condition d:

$$y_{id} = \beta_0 + \beta_1 x_{id} + \theta_{\text{char}(i)} + e_i.$$

Here y_{id} is the outcome from Day 2 plotted on the y-axis and x_{id} is the corresponding measure from Day 1 plotted on the x-axis.

Total Completion Time model with participant characteristics as controls. Panel C of Table S3 reports the Total Completion Time model that includes participant characteristics as controls, with Human Confirmation as the reference group:

$$y_{it} = \beta_0 + \beta_1 \text{Creativity}_i + \beta_2 \text{Copilot}_i + \beta_3 \text{Post}_t + \beta_4 (\text{Creativity}_i \times \text{Post}_t) + \beta_5 (\text{Copilot}_i \times \text{Post}_t) + \theta_{\text{char}(i)} + e_{it}.$$

Here Post_t indicates Day 2, Creativity_i indicates Human Creativity, and Copilot_i indicates Copilot; Human Confirmation is the omitted reference group.

Two-wave model with participant fixed effects. For outcomes measured on both days, this specification uses a fixed effect for each participant instead of separate controls for participant characteristics. For completion time in Panel A of Table S3, the model is

$$y_{it} = \alpha_i + \tau \text{Post}_t + \delta_1 (\text{Confirm}_i \times \text{Post}_t) + \delta_2 (\text{Copilot}_i \times \text{Post}_t) + \varepsilon_{it}.$$

The coefficient τ is the change from Day 1 to Day 2 for the Human Creativity reference group. δ_1 and δ_2 estimate how the Day 2 minus Day 1 change differs for Human Confirmation and Copilot, respectively, relative to Human Creativity.

For story quality, Table S14 reports two fixed-effects specifications. The rating-level model uses evaluator-story ratings:

$$q_{irt}^{(m)} = \alpha_i + \tau^{(m)} \text{Post}_t + \delta_1^{(m)} (\text{Confirm}_i \times \text{Post}_t) + \delta_2^{(m)} (\text{Copilot}_i \times \text{Post}_t) + \varepsilon_{irt}^{(m)},$$

where $q_{irt}^{(m)}$ is evaluator r's rating of participant i's story on day t for quality dimension m. The participant-day model first averages evaluator ratings to the participant-day level and then estimates

$$\bar{q}_{it}^{(m)} = \alpha_i + \tau^{(m)} \text{Post}_t + \delta_1^{(m)} (\text{Confirm}_i \times \text{Post}_t) + \delta_2^{(m)} (\text{Copilot}_i \times \text{Post}_t) + \bar{\varepsilon}_{it}^{(m)}.$$

For process satisfaction, Table S17 uses the same two-wave model with participant fixed effects for each satisfaction item m:

$$s_{it}^{(m)} = \alpha_i + \tau^{(m)} \text{Post}_t + \delta_1^{(m)} (\text{Confirm}_i \times \text{Post}_t) + \delta_2^{(m)} (\text{Copilot}_i \times \text{Post}_t) + \varepsilon_{it}^{(m)}.$$

Paired tests within each collaboration design. Tables S3, S15, and S16 also report Day 2 minus Day 1 changes within each collaboration design. For each participant with both observations from both days, we compute

$$\Delta y_i = y_{i2} - y_{i1}$$

and test whether the mean within-group change differs from zero using paired t-tests.

B Materials

B.1 Day 1 Instruction

Participants received the following Day 1 instructions for writing a 1,000-word short story without ChatGPT.

1. **Verify ID and group number.** Participants checked their ID and group number in the Google spreadsheet.
2. **Take the DAT creativity test.** Participants completed the DAT creativity test, kept their score for the survey, and were asked to take the test in a distraction-free environment.
3. **Complete the initial survey.** Participants completed the baseline survey before beginning the writing task.
4. **Duplicate and rename the submission template.** Participants copied the Google document template, renamed the file to include the day, ID, and group number (for example, "Day1-7A" or "Day1-18B"), and uploaded the document to Canvas under the Day 1 assignment.
5. **Write the short story without AI support.** Participants were asked to write a 1,000-word story manually. The story could be in any genre and should be original, interesting, coherent, clear, comprehensible, and high quality. Participants could use the narrative structure guide in Section B.2.4 but did not have to follow it exactly. They were instructed not to use ChatGPT or any AI support and not to discuss the assignment with classmates.
6. **Follow the writing process.** Participants recorded when they began the assignment and followed four writing steps: ideation, outlining, writing, and editing.
7. **Submit the story and exit survey.** Participants uploaded the completed story to Canvas under "Experiment Day 1" and completed the exit survey.

B.2 Day 2 Instruction

All Day 2 groups received the same opening instructions for writing a 1,000-word short story with ChatGPT.

1. **Verify ID and group number.** Participants checked their ID and group number in the Google spreadsheet.
2. **Duplicate and rename the submission template.** Participants copied the Google document template and renamed the file to include the day, ID, and group number (for example, "Day2-7A").
3. **Write the short story with ChatGPT.** Participants were asked to write a 1,000-word story in collaboration with ChatGPT. The story could be in any genre and should be original, interesting, coherent, clear, comprehensible, and high quality. Participants could use the narrative structure guide in Section B.2.4 but did not have to follow it exactly. They were instructed to review the prompt-engineering guidelines from class and use ChatGPT only as described for their group.
4. **Submit the story and exit survey.** Participants uploaded the completed story to the assignment section labeled "Experiment Day 2" and completed the exit survey.

The common writing process included ideation, outlining, writing, and editing. The assigned collaboration design determined which parts participants completed themselves and which parts they assigned to ChatGPT.

B.2.1 Human Confirmation

In Human Confirmation, ChatGPT handled ideation, outlining, writing, and editing. Participants assigned tasks to ChatGPT through prompts, evaluated its work, and either approved the output or asked ChatGPT to try again. They were instructed not to offer additional ideas to ChatGPT during any stage of the process.

- **Ideation.** Participants asked ChatGPT to generate story ideas, reviewed the ideas, selected one, or asked for additional options.
- **Outlining.** Participants asked ChatGPT to create an outline for the chosen story idea using the seven-sequence story structure.
- **Writing.** Participants asked ChatGPT to expand the approved outline into a full 1,000-word story, either section by section or all at once. If dissatisfied, they could ask ChatGPT to rewrite the text.
- **Editing.** Participants asked ChatGPT to edit the story for readability, tone, flow, or a specific writing style until they were satisfied with the final story.

B.2.2 Human Creativity

In Human Creativity, participants completed ideation and outlining manually. ChatGPT then handled writing and editing based on the participants' prompts. Participants were instructed not to offer additional ideas to ChatGPT during the writing and editing stages.

- **Ideation.** Participants generated one or more story ideas without ChatGPT and selected the idea they wanted to use.
- **Outlining.** Participants wrote an outline manually, roughly following the seven-sequence story structure.
- **Writing.** Participants prompted ChatGPT to expand their manually created outline into a full 1,000-word story, either section by section or all at once. They could ask for rewrites if dissatisfied.
- **Editing.** Participants asked ChatGPT to edit the story for readability, tone, flow, or a specific writing style until they were satisfied with the final story.

B.2.3 Copilot

In Copilot, participants could collaborate with ChatGPT throughout ideation, outlining, writing, and editing. They could begin from their own ideas and use ChatGPT for feedback, ask ChatGPT to start the task, or move iteratively between their own writing and ChatGPT's suggestions.

- **Ideation.** Participants could share their own story ideas with ChatGPT for suggestions, ask ChatGPT to generate story ideas, or combine elements from different suggestions.
- **Outlining.** Participants could ask ChatGPT to generate an initial outline and refine it, or create an outline manually and ask ChatGPT for additional suggestions.
- **Writing.** Participants could write sections themselves, ask ChatGPT to fill in parts, or ask ChatGPT to write the story and then provide feedback for revisions.
- **Editing.** Participants could ask ChatGPT to suggest edits, edit the text themselves, or provide specific feedback on preferred edits until they were satisfied with the final story.

B.2.4 Narrative Structure Guide

Participants were told that, if they did not have experience with creative writing or felt time-constrained, they could use the seven-sequence story structure from ScriptLab. The guide was optional, and participants could modify it as needed.

1. **Sequence One: Status Quo and Inciting Incident.** Establish the central character, the character's life, and the world of the story. This sequence usually ends with the point of attack or inciting incident.
2. **Sequence Two: Predicament and Lock In.** Set up the central predicament and the first hint of obstacles. The sequence ends when the main character is locked into the predicament and pushed in a new direction.
3. **Sequence Three: First Obstacle and Raising the Stakes.** Show the first obstacle and the alternatives available to the character. As these alternatives are eliminated, the stakes increase.
4. **Sequence Four: First Culmination or Midpoint.** Reach the midpoint of the story, where the character faces a higher obstacle and the story builds to a first culmination.
5. **Sequence Five: Subplot and Rising Action.** Introduce or develop a subplot that becomes more important to the story.
6. **Sequence Six: Main Culmination or End of Act Two.** Build toward the main culmination and the lowest moment, or highest moment in a tragedy, before the final act.
7. **Sequence Seven: Resolution.** Bring the story to its conclusion. The guide encouraged a resolution with clarity and, when appropriate, a twist that is surprising but inevitable.

B.3 Survey

B.3.1 Initial Survey on Day 1

Experiment Day 1: Initial Survey. Please complete the initial survey to provide baseline data prior to starting the experiment. Rest assured that your responses to this survey will not impact your score in any manner, so we encourage you to answer honestly.

Q1. Name, ID, and Group

Q2. Rate your comfort with the English language. Native communicator (speaking and writing in English since early childhood); Not-native but fluent in English; Not fluent in English

Q3. Rate your level of experience with creative writing. Some professional or academic experience in creative writing; No professional experience but ample creative writing experience in school, college, and beyond; Limited exposure to creative writing

Q4. Rate your creative writing ability relative to your peers. Above average; Average; Below Average

Q5. Please write down your 'DAT creativity test' score here.

B.3.2 DAT Score Survey

The Divergent Association Task (DAT) was conducted through the website `https://www.datcreativity.com/`. Participants were provided with consent information on the site before proceeding to write 10 different words that were mostly unrelated to each other. The test typically takes 2 to 4 minutes to complete. After completing the task, participants were asked to record their DAT Score on the initial survey on Day 1. We informed participants that they could take the test only once and instructed them to report the score from their first attempt.

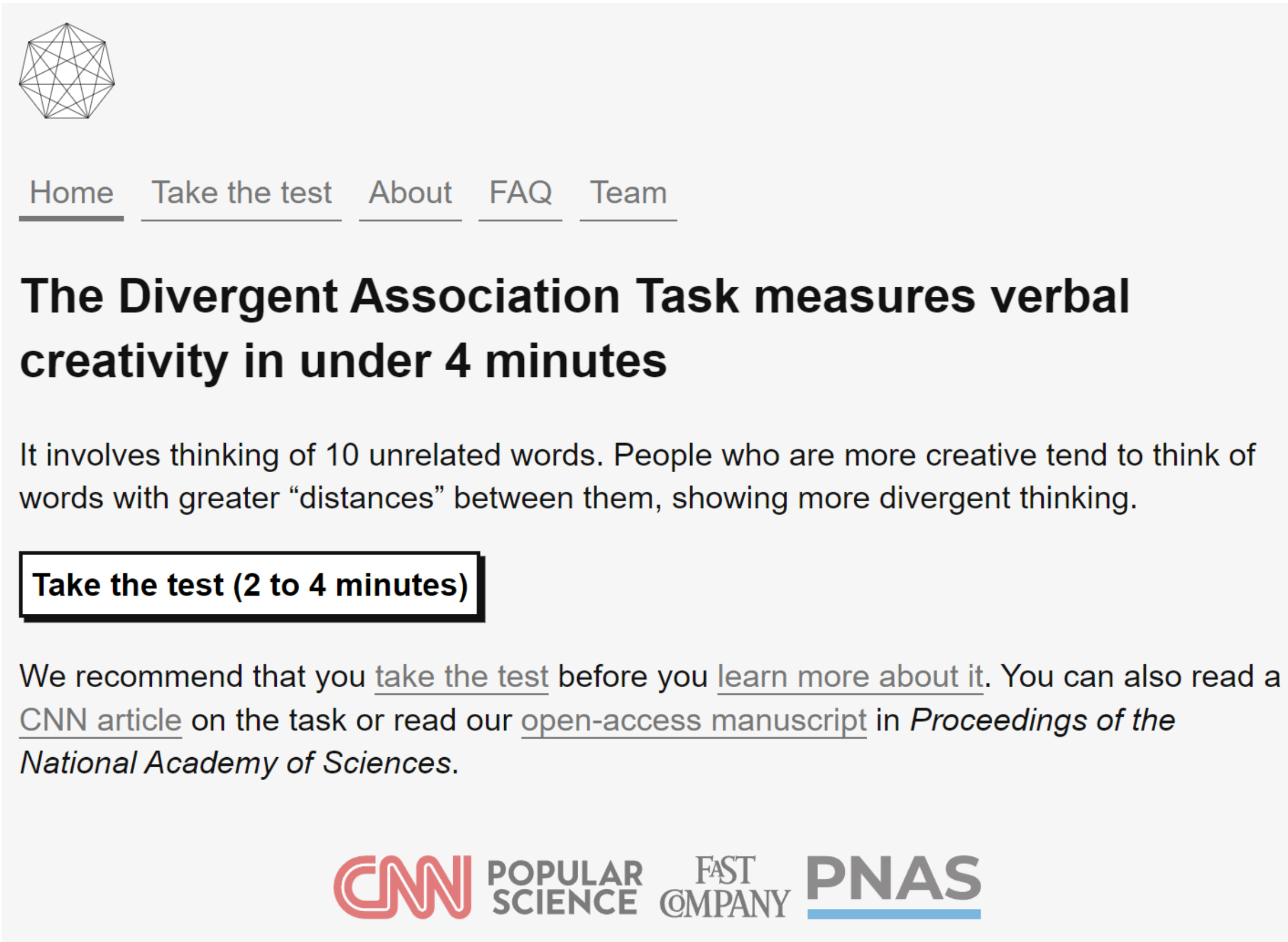
Home Take the test About FAQ Team

The Divergent Association Task measures verbal creativity in under 4 minutes

It involves thinking of 10 unrelated words. People who are more creative tend to think of words with greater “distances” between them, showing more divergent thinking.

Take the test (2 to 4 minutes)

We recommend that you take the test before you learn more about it. You can also read a CNN article on the task or read our open-access manuscript in *Proceedings of the National Academy of Sciences*.

CNN POPULAR SCIENCE FAST COMPANY PNAS

Reproduced DAT website screen: task overview.

Instructions

Please enter 10 words that are as **different** from each other as possible, in all meanings and uses of the words.

Rules

1. Only **single words** in English.
2. Only **nouns** (e.g., things, objects, concepts).
3. **No proper nouns** (e.g., no specific people or places).
4. **No specialised vocabulary** (e.g., no technical terms).
5. Think of the words **on your own** (e.g., do not just look at objects in your surroundings).

Consent

Contribute your anonymous responses to our research?

○ Yes
○ No

▼ **Study details**

In this 4-minute survey, you will be asked to complete a creativity task and report your age, gender, and country. Participating is voluntary, there are no foreseeable risks, and your responses remain anonymous. The study will help researchers understand creativity across the world.

For study questions, contact Dr. Jay Olson. For questions about your rights as a research participant, contact the Research Oversight and Compliance Office—Human Research Ethics Program at ethics.review@utoronto.ca.

— Dr. Jay Olson and Dr. Loren Martin
University of Toronto Mississauga

Reproduced DAT website screen: instructions and consent.

Enter words

1. Enter a single noun
2. Enter a single noun
3. Enter a single noun
4. Enter a single noun
5. Enter a single noun
6. Enter a single noun
7. Enter a single noun
8. Enter a single noun
9. Enter a single noun
10. Enter a single noun

Demographics (optional)

Age:

Sex: (none)

Country: (none)

Bonus question (optional)

One hears about “morning types” and “evening types.” Which one of these types do you consider yourself to be?

- ○ Definitely a morning type
- ○ Rather more a morning type than an evening type
- ○ Rather more an evening type than a morning type
- ○ Definitely an evening type

Submit

Reproduced DAT website screen: word-entry form.

B.3.3 Exit Survey on Day 1

Q1. Name, ID, and Group
Q2. Enter the Number of Minutes to Complete the Full Assignment, including brainstorming, outlining, writing, and editing.
Q3. Enter the Number of Minutes Spent on Brainstorming.
Q4. Enter the Number of Minutes Spent on Outlining.
Q5. Enter the Number of Minutes Spent on Writing.
Q6. Enter the Number of Minutes Spent on Editing.
Q7. How would you rate your overall satisfaction with the writing process? Extremely dissatisfied (1); Moderately dissatisfied (2); Slightly dissatisfied (3); Neither satisfied nor dissatisfied (4); Slightly satisfied (5); Moderately satisfied (6); Extremely satisfied (7)
Q8. Did the writing process allow for flexibility to achieve what you wanted? Extremely disagree (1); Moderately disagree (2); Slightly disagree (3); Neither agree nor disagree (4); Slightly agree (5); Moderately agree (6); Extremely agree (7)
Q9. Do you feel confident that you will be able to achieve your writing goals with this process? Extremely disagree (1); Moderately disagree (2); Slightly disagree (3); Neither agree nor disagree (4); Slightly agree (5); Moderately agree (6); Extremely agree (7)
Q10. Did the writing process help to achieve what you wanted? Would you use the same writing process again in the future?
No; Yes
Q11. [If answer was No]: What would you change?

B.3.4 Exit Survey on Day 2

Q1. Name, ID, and Group
Q2. Enter the Number of Minutes to Complete the Full Assignment, including brainstorming, outlining, writing, and editing.
Q3. Enter the Number of Minutes Spent on Brainstorming.
Q4. Enter the Number of Minutes Spent on Outlining.
Q5. Enter the Number of Minutes Spent on Writing.
Q6. Enter the Number of Minutes Spent on Editing.
Q7. How would you rate your overall satisfaction with the writing process? Extremely dissatisfied (1); Moderately dissatisfied (2); Slightly dissatisfied (3); Neither satisfied nor dissatisfied (4); Slightly satisfied (5); Moderately satisfied (6); Extremely satisfied (7)
Q8. Did the writing process allow for flexibility to achieve what you wanted? Extremely disagree (1); Moderately disagree (2); Slightly disagree (3); Neither agree nor disagree (4); Slightly agree (5); Moderately agree (6); Extremely agree (7)
Q9. Do you feel confident that you will be able to achieve your writing goals with this process? Extremely disagree (1); Moderately disagree (2); Slightly disagree (3); Neither agree nor disagree (4); Slightly agree (5); Moderately agree (6); Extremely agree (7)
Q10. Did the writing process help to achieve what you wanted? Would you use the same writing process again in the future?
No; Yes
Q11. [If answer was No]: What would you change?
Q12. How satisfied are you with the AI's help in your writing, including the quality of its suggestions and how much it boosted your productivity? Extremely dissatisfied (1); Moderately dissatisfied (2); Slightly dissatisfied (3); Neither satisfied nor dissatisfied (4); Slightly satisfied (5); Moderately satisfied (6); Extremely satisfied (7)
Q13. How satisfied were you with the AI-human collaboration experience you were asked to follow? Extremely dissatisfied (1); Moderately dissatisfied (2); Slightly dissatisfied (3); Neither satisfied nor dissatisfied (4); Slightly satisfied (5); Moderately satisfied (6); Extremely satisfied (7)
Q14. Please specify ChatGPT's contribution to generating the story, using a scale from 0% to 100% (where 0% indicates you contributed 100%, 100% indicates ChatGPT contributed 100%, and 50% indicates an equal contribution from both).

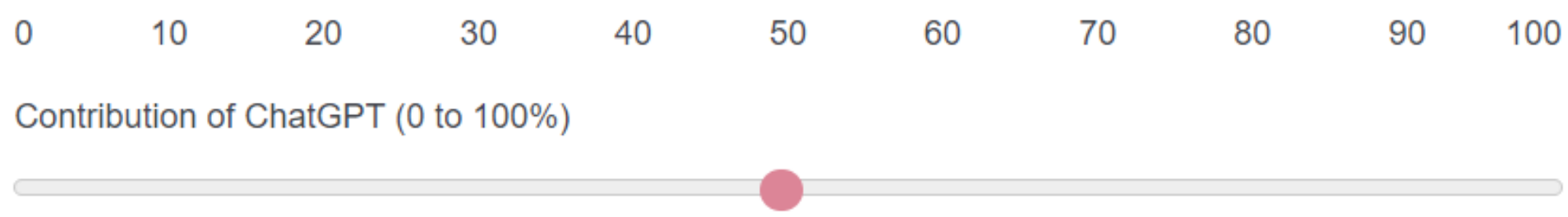

B.4 Evaluation

Q1. Name, ID, and Group
Q2. Please enter the File Name (e.g., 747B9xv8XAViqblr.docx) of the paper you are reviewing.
Q3. [Originality] Is the story original and creative? For example, does the story use novel themes, original plot/setting, unusual story structure, unusual ending, and surprising and unexpected style and emotional tone? Extremely unoriginal (1); Moderately unoriginal (2); Slightly unoriginal (3); Neither original nor unoriginal (4); Slightly original (5); Moderately original (6); Extremely original (7)
Q4. [Interestingness] Does the story provide an interesting and immersive experience for readers, engaging them deeply? For example, does the story use literary devices to convey vivid images and scenes to engage readers? Extremely uninteresting (1); Moderately uninteresting (2); Slightly uninteresting (3); Neither interesting nor uninteresting (4); Slightly interesting (5); Moderately interesting (6); Extremely interesting (7)
Q5. [Writing Quality] Is the language clear, comprehensible, and easy to understand? In addition, is the text free of errors in grammar, punctuation, and spelling? Extremely poor writing quality (1); Moderately poor writing quality (2); Slightly poor writing quality (3); Neither good nor poor writing quality (4); Slightly good writing quality (5); Moderately good writing quality (6); Extremely good writing quality (7)

Q6. [Coherence] Is the story well-structured and organized? Extremely poor coherence (1); Moderately poor coherence (2); Slightly poor coherence (3); Neither good nor poor coherence (4); Slightly good coherence (5); Moderately good coherence (6); Extremely good coherence (7)

Q7. [Overall Quality] What is your overall assessment of the story? For example, does the story effectively convey its main ideas in a manner that is creative, interesting, well-written, and coherent? Extremely bad (1); Moderately bad (2); Slightly bad (3); Neither good nor bad (4); Slightly good (5); Moderately good (6); Extremely good (7)

C Supplementary Text

C.1 Reflective Feedback Analysis

We analyze participants' qualitative feedback about their experiences with AI-assisted writing. Participants wrote reflections on what worked, what did not, and what they would change. These reflections were collected from 270 participants. Following the method outlined by Ludan *et al.* (16), we used large language models (LLMs) to identify ten positive and ten negative topics across the full set. We then used Claude Sonnet 4.6 to classify each participant's reflection into those topics. Reflections could be assigned to none, one, or multiple topics.

Differences across Collaboration Designs. The qualitative feedback gives context for differences across collaboration designs, especially the lower perceived flexibility and reduced sense of ownership reported by participants in the Human Confirmation group.

In the Human Confirmation group, participants mainly confirmed AI-generated outputs, and several described feeling less involved in the creative process. This reduced involvement was a source of frustration and disconnection in their comments. For instance, one student mentioned feeling "super removed from the content of the story," which led to a final product they were "far from proud of." This disengagement contributed to a reduced sense of ownership over the final story, as students felt disconnected from the creative parts of the work. Moreover, students in this group often struggled with the AI's narrative direction. They reported that AI outputs frequently "diverged" from their creative vision, making it challenging to produce a coherent and satisfying narrative. One student expressed, "I struggled to redirect the storyline without giving it ideas/examples/nudges," describing how the AI's limits in nuanced storytelling left the narrative feeling "generic" and unaligned with their vision. The AI's difficulty with subtle details and stylistic elements resulted in stories that felt "flat" and lacked depth. These comments help explain why the Human Confirmation process felt less flexible and less satisfying to many participants.

In contrast, students in the Human Creativity group experienced a greater sense of creative control, which enabled them to maintain a stronger connection to the story. As one student noted, being responsible for the story's ideation and outlining allowed them to "keep the story aligned with my vision from the start," leading to a more satisfying experience. However, while students appreciated the creative control, they also encountered challenges with AI's execution. The need for frequent adjustments and prompts to achieve the desired narrative showed the limits of AI in realizing the students' creative intentions. For instance, one student noted, "I couldn't get the AI story to be exactly the way I wanted it. It just didn't have my voice, and I had a hard time infusing my voice into its system," describing the challenge of making the AI's output match their creative vision. Despite these challenges, active involvement in the creative process supported a greater sense of ownership and a more positive experience of the writing process.

Similarly, participants in the Copilot group worked with AI throughout all stages of the writing process and often described the process as flexible and collaborative. Students in this group could either initiate tasks or use AI for feedback and refinement. One student described the experience as a "true partnership," where the AI was used to enhance rather than replace their creative input. This iterative and collaborative approach helped students retain creative control while refining the narrative. For example, one student noted, "The iterative process of suggesting tweaks for each paragraph and then asking ChatGPT to rewrite it was particularly insightful," which describes a process in which the student remained involved while using AI to revise the story.

The qualitative feedback suggests that participants described the process more positively when they retained creative control and could decide how to use AI support.

Opportunities in Writing with AI. Table S1 summarizes ten opportunities that participants described when writing with large language model support, grouped into three areas. In *Efficiency and Workflow Enhancement*, participants described AI as helping them maintain structure, reduce writing time, and plan the story. In *Creativity and Idea Generation*, participants described AI as generating varied storylines and supporting ideation and revision. In *Language and Structural Support*, participants described help with grammar, coherence, research, and fact-checking.

Challenges in Writing with AI. Table S2 summarizes ten challenges that participants described when writing with large language model support, grouped into three areas. In *Authenticity and Emotional Depth*, participants described AI responses as sometimes lacking a human touch, emotional depth, or authenticity. In *Technical and Logical Consistency*, participants described difficulty getting AI to follow complex instructions and maintain a consistent narrative voice. In *Creativity and Critical Thinking*, participants described limitations in generating novel ideas and in handling humor or irony. These comments identify tasks where participants still wanted human judgment.

Coded analysis of the reflection essays. We further coded the same reflection essays to examine how the collaboration designs differed in the writing process participants described and in their reported experience of the final story and AI collaboration. Two features of the designs could plausibly explain why Human Confirmation performed worst while Human Creativity and Copilot were similar on many outcomes. One is that excluding participants from early ideation and outlining weakens their ownership of the work. The other is greater difficulty in carrying personal voice and narrative coherence into the AI's text. The coding examines both. The reflection prompt asked participants to describe their writing process and how it felt (SI Section A.1), so both are grounded in participants' own narration. A large language model (Claude Sonnet 4.6), separate from the assistance participants

used while writing, coded each essay without the assigned condition, which was joined only afterward. We coded what participants said they did, and treated a code as absent when it was only hypothetical, recommended for future work, or present only inside AI text the participant had pasted in. Two passes were used: a process pass of binary indicators of who did each step (Table S19), and an experience pass rating ten themes from 0 (absent) to 3 (strong). Coherence appears in both passes, once as whether a problem was mentioned and once as how strongly it was expressed. Because a single model produced the codes, we report the experience codes alongside, and check them against, the participants' own Day-2 survey responses.

Process ownership: where creative control sat. The first form of ownership is over the story-making process—whether the participant actually shaped how the story was made. The process codes show where that control sat; they describe how the designs were enacted, not a causal mechanism. The designs differed sharply at the early stages and not at drafting (Table S20). Under Human Confirmation the AI produced the initial idea in 80.2% of essays and the participant in only 2.2%, and the AI produced the outline in 54.9%. Under Human Creativity participants produced the idea and the outline themselves in 77.7% of essays. Copilot also used the AI during ideation (an AI-produced idea in 57.6% of essays), but participants remained involved through final editing in 69.4% and substantial rewriting in 28.2%. AI drafting and iterative prompting were common in all three designs and did not differ. The designs therefore differ not in whether the AI drafted, but in where participants contributed to the creative work: during ideation and outlining under Human Creativity, through rewriting and final editing under Copilot, and primarily through prompting and approval under Human Confirmation. Process ownership is thus weakest under Human Confirmation and preserved, in different forms, under Human Creativity and Copilot.

Voice and coherence. We first examined whether Human Confirmation made it harder to inject personal voice and narrative coherence. Voice or style mismatch did not differ across designs (process pass $p = .53$; the experience-pass voice code also did not differ, $p = .74$). Coherence problems were less common under Human Creativity than under Human Confirmation ($p = .003$), but Human Confirmation and Copilot did not differ ($p = .76$); coherence therefore does not track the Human Confirmation deficit. The quality differences themselves are established by the rater-based results in the main text; these self-reports do not add a separate quality mechanism, and we do not foreground voice or coherence as the reason Human Confirmation underperforms.

Output ownership and collaboration. The second form of ownership is over the finished story—whether the final product felt like the participant's own. Its most direct positive marker, pride in the story, was lower under Human Confirmation than under Human Creativity and Copilot combined ($p = .0052$, nominal). Explicit markers of lost ownership—feeling the output was not one's own, being reduced to approving the AI's choices, or having one's voice suppressed—did not differ across designs (Table S21), so the output-ownership difference shows as lower pride rather than as voiced complaints of lost authorship. Participants under Human Confirmation also reported less partnership with the AI ($p = .0025$, surviving a Bonferroni correction across the ten themes), a collaborative experience—feeling like a genuine co-creator rather than an approver—that we keep distinct from ownership of the output. Human Creativity and Copilot did not differ on pride or partnership ($p = .28$, $p = .11$). The output-ownership signal is the contrast between Human Confirmation and Human Creativity and Copilot combined, not a difference between Human Creativity and Copilot. We label these "expressed pride" and "expressed partnership" to distinguish the coded reactions from the survey measures; they converged with participants' own Day-2 survey satisfaction and flexibility (Spearman $\rho = .24$ to $.33$) and were unrelated to externally rated story quality ($|\rho| \leq .05$). Controlling for Day-2 story quality left the Human Confirmation gaps essentially unchanged, so they are not simply a reaction to having produced a weaker story.

Interpretation. Taken together, the reflections separate two forms of ownership. Process ownership—shaping how the story was made—differs most clearly: Human Confirmation cedes the initial idea and outline to the AI, Human Creativity preserves human control during ideation and outlining, and Copilot preserves human involvement through rewriting and final editing. Output ownership—feeling the finished story is one's own—is lower under Human Confirmation, seen in reduced pride rather than in explicit complaints of lost authorship. Their lower partnership with the AI is a separate collaborative experience, not a further measure of ownership. This sharpens the qualitative reflective-feedback reading that Human Confirmation weakens ownership: the weakening appears through where creative control sat and in reduced pride, not through voiced loss. It is consistent with the ownership/IKEA-effect account in the main text—contributing to the creative work, whether during ideation and outlining or throughout the writing process, coincides with a stronger sense of having produced one's own story—and we present it as process and experience evidence consistent with the outcome differences, not as a causal test.

C.2 Other Tables and Figures

Changes in Total Completion Time.

Table S3 reports three analyses of Total Completion Time. Panel A uses a model with participant fixed effects to estimate Day 2 minus Day 1 changes and contrasts across collaboration designs. Panel B reports paired changes for the same participants. Panel C reports a model with participant characteristics as controls. Across all three collaboration designs, participants spent less time on Day 2 than on Day 1. The across-design interaction estimates are small relative to these within-person changes and have wide standard errors.

Differences in Additional Quality Metrics.

Tables S4 and S7 report Day 2 quality comparisons with participant characteristics included as controls. Table S4 reports pairwise comparisons for Originality, Interestingness, Writing Quality, and Coherence; positive pairwise coefficients indicate higher adjusted Day 2 ratings for the design listed before "minus." Table S7 reports Overall Quality comparisons by collaboration design, with Human Confirmation as the reference design for the displayed treatment coefficients.

In these Day 2 level analyses, Human Creativity and Copilot have higher adjusted Overall Quality than Human Confirmation, and they have higher adjusted Day 2 values than Human Confirmation on several of the four Table S4 quality ratings. Tables S14 and S15 report changes from Day 1 to Day 2, not Day 2 level comparisons: Table S14 reports change estimates from models with participant fixed effects, and Table S15 reports paired within-person changes in story quality.

Association of Additional Quality Metrics with Overall Quality. Table S5 reports how Originality, Interestingness, Writing Quality, and Coherence are associated with Overall Quality. The regression is estimated at the level of individual evaluator ratings. Interestingness has the largest association with Overall Quality, followed by Coherence, Originality, and Writing Quality.

Completion Time and Quality Analyses. Tables S6-S9 report additional analyses of completion time and Overall Quality. Tables S6 and S8 ask whether differences in the Day 1 outcome persist in the corresponding Day 2 outcome within each collaboration design. Coefficients closer to 1 indicate stronger persistence, whereas coefficients closer to 0 indicate weaker persistence. Tables S6-S8 each include specifications with and without controls for participant characteristics. Table S9 reports the association between DAT Score and Overall Quality separately for Day 1 and Day 2; its second specification adds Creative Writing Experience, Creative Writing Ability, and English Language Proficiency to the DAT Score specification.

Supplementary Satisfaction Analyses. Table S10 reports Day 2 minus Day 1 changes in Process Satisfaction, Flexibility, Process Effectiveness, and Intent to Reuse Process within each collaboration design. Table S11 reports Day 2 comparisons across collaboration designs for the same four process satisfaction measures. Table S12 reports Day 2 comparisons across collaboration designs for AI Effectiveness, Satisfaction with AI, and AI's Contribution. Tables S16 and S17 report within-person changes and pairwise change contrasts, including direct Human Creativity versus Copilot contrasts.

On Day 2, Human Confirmation had lower Process Satisfaction than Human Creativity and Copilot. In the paired within-person tests, Flexibility declined under Human Confirmation, while Human Creativity and Copilot improved on several other process items. In Table S17, the change contrasts with participant fixed effects involving Human Confirmation are negative but not statistically distinguishable from zero.

Independent Genre Classification. Table S18 compares GPT-4o and Qwen2.5-72B-Instruct classifications for the same story records under the same nine-genre multi-label scheme. The agreement measures and prevalence correlations show how sensitive the group-level genre patterns are to the classification model. The genre-specific prevalence estimates should be read with caution because some prevalence differences between the two classification models are large.

D Supplementary Tables and Figures

Figure S0. Participant Counts and Submitted Story Records

Enrolled and randomized
$N = 285$
Human Creativity = 99; Human Confirmation = 95; Copilot = 91

$\Downarrow$

Day 1 story submissions
$N = 278$
Human Creativity = 96; Human Confirmation = 93; Copilot = 89

$\Downarrow$

Day 2 story submissions
$N = 271$
Human Creativity = 93; Human Confirmation = 88; Copilot = 90

$\Downarrow$

Stories submitted across Days 1 and 2
$N = 549$

Note: Figure S0 separates participant counts from submitted story records. The enrollment box counts randomized participants. The Day 1 and Day 2 boxes count submitted stories for those writing days; because each participant could submit at most one story per day, those counts also correspond to the participants who submitted a story on that day. The final box counts story records across both writing days: 278 Day 1 stories plus 271 Day 2 stories, for 549 story records. Table S0a reports the participant samples, story records, evaluator ratings, survey responses, participants with written reflective feedback, and story records used in genre classification in the supplementary tables.

Table S0a. Counts for Participants, Stories, Ratings, Surveys, and Genre Analysis

Sample or record set	Count	Definition and use
Enrolled and randomized participants	285	Participants randomized to a Day 2 collaboration design. Randomized group counts were Human Creativity = 99, Human Confirmation = 95, and Copilot = 91.
All four participant characteristics	280	Participants reported Creative Writing Experience, Creative Writing Ability, and English Language Proficiency and completed the Divergent Association Task (DAT) before Day 2. These four variables are the controls in models that adjust for participant characteristics. Group counts were Human Creativity = 97, Human Confirmation = 92, and Copilot = 91.
Four participant characteristics plus ten Day 1 outcomes	256	This sample includes the four participant characteristics listed above and ten Day 1 outcomes: Overall Quality, Originality, Interestingness, Writing Quality, Coherence, Process Satisfaction, Flexibility, Process Effectiveness, Intent to Reuse Process, and Day 1 Total Completion Time. It is used for balance checks that keep the same participants across all variables and for the joint balance model. Group counts were Human Creativity = 91, Human Confirmation = 80, and Copilot = 85.
Day 1 story records	278	Stories submitted on Day 1, when participants wrote without AI. Group counts were Human Creativity = 96, Human Confirmation = 93, and Copilot = 89.
Day 2 story records	271	Stories submitted on Day 2, when participants wrote with AI under their assigned collaboration design. Group counts were Human Creativity = 93, Human Confirmation = 88, and Copilot = 90.
All submitted story records	549	Day 1 story records plus Day 2 story records.
Evaluator ratings of story quality	2,196	Four independent evaluator ratings for each submitted story record.
Matched story quality sample	264	Participants had story quality outcomes on both days for paired story quality analyses. Group counts were Human Creativity = 90, Human Confirmation = 86, and Copilot = 88.
Matched Total Completion Time sample	230	Participants reported Total Completion Time on both days for models with participant fixed effects and paired analyses of Total Completion Time. Group counts were Human Creativity = 84, Human Confirmation = 68, and Copilot = 78.
Any process satisfaction report	276	Participants reported at least one process satisfaction outcome on Day 1 or Day 2.
Process satisfaction reports across Days 1 and 2	508	Day 1 and Day 2 process satisfaction reports from participants with at least one process satisfaction report.
Matched process satisfaction sample	232	Participants reported process satisfaction outcomes on both days for paired process satisfaction analyses.
Day 2 ratings about AI assistance	248	Participants reported AI Effectiveness, Satisfaction with AI, and AI's Contribution on Day 2.
Written reflective feedback	270	Participants provided open-ended reflective feedback.
Story records for genre analysis	557	Story records classified for the genre analysis reported in Figure 4 and Table S18. This record set contains eight more records than the 549 story records used in the quality analyses. Group counts were Day 1 = 282 and Day 2 Human Creativity = 93, Human Confirmation = 91, and Copilot = 91.

Note: Rows mix units: participants, story records, evaluator ratings, survey reports counted by participant and day, written feedback, and story records classified by genre. Counts should not be added across rows. Missing story submissions, surveys, or later measures cause the analysis samples to differ across outcomes.

Table S0b. Baseline Balance Across Randomized Groups

Panel A. Baseline means by randomized group

Variable	Human Creativity	Human Confirmation	Copilot
DAT Score	97 / 81.32 (9.04)	92 / 82.58 (6.26)	91 / 83.00 (5.38)
Creative Writing Experience	97 / 1.65 (0.75)	92 / 1.52 (0.65)	91 / 1.69 (0.73)
Creative Writing Ability	97 / 1.94 (0.66)	92 / 1.82 (0.69)	91 / 1.93 (0.66)
English Language Proficiency	97 / 2.85 (0.36)	92 / 2.71 (0.46)	91 / 2.75 (0.46)
Day 1 Overall Quality	96 / 4.90 (0.77)	93 / 4.92 (0.72)	89 / 4.77 (0.77)
Day 1 Originality	96 / 4.93 (0.94)	93 / 4.94 (0.79)	89 / 4.84 (0.94)
Day 1 Interestingness	96 / 4.91 (0.86)	93 / 4.96 (0.82)	89 / 4.70 (0.86)
Day 1 Writing Quality	96 / 4.99 (0.85)	93 / 5.04 (0.79)	89 / 4.86 (0.89)
Day 1 Coherence	96 / 5.19 (0.79)	93 / 5.15 (0.82)	89 / 5.03 (0.72)
Day 1 Process Satisfaction	92 / 4.52 (1.54)	83 / 4.69 (1.42)	85 / 4.85 (1.29)
Day 1 Flexibility	92 / 5.01 (1.29)	83 / 5.05 (1.29)	85 / 5.09 (1.37)
Day 1 Process Effectiveness	92 / 4.33 (1.53)	83 / 4.45 (1.52)	85 / 4.55 (1.36)
Day 1 Intent to Reuse Process	92 / 0.64 (0.48)	83 / 0.73 (0.44)	85 / 0.68 (0.47)
Day 1 Total Completion Time	92 / 70.03 (21.75)	81 / 72.33 (35.62)	85 / 68.24 (14.91)

Panel B1. Omnibus tests of baseline balance

Variable	Row-specific sample F	Row-specific sample p	Same 256 participants F	Same 256 participants p
DAT Score	1.42	0.244	1.29	0.277
Creative Writing Experience	1.43	0.242	1.41	0.246
Creative Writing Ability	1.00	0.369	0.61	0.546
English Language Proficiency	2.63	0.074	2.57	0.078
Day 1 Overall Quality	1.10	0.333	1.20	0.302
Day 1 Originality	0.36	0.700	0.95	0.387
Day 1 Interestingness	2.49	0.085	3.47	0.033
Day 1 Writing Quality	1.07	0.346	0.89	0.411
Day 1 Coherence	1.06	0.348	0.77	0.466
Day 1 Process Satisfaction	1.16	0.316	1.31	0.272
Day 1 Flexibility	0.09	0.915	0.07	0.936
Day 1 Process Effectiveness	0.53	0.592	0.55	0.576
Day 1 Intent to Reuse Process	0.88	0.415	1.05	0.351
Day 1 Total Completion Time	0.55	0.580	0.58	0.561

Panel B2. Pairwise Welch test *p*-values

Variable	Human Creativity–Human Confirmation	Human Creativity–Copilot	Human Confirmation–Copilot
DAT Score	0.267	0.122	0.627
Creative Writing Experience	0.213	0.691	0.097
Creative Writing Ability	0.214	0.966	0.238
English Language Proficiency	0.023	0.109	0.550
Day 1 Overall Quality	0.875	0.236	0.172
Day 1 Originality	0.947	0.504	0.434
Day 1 Interestingness	0.662	0.096	0.035
Day 1 Writing Quality	0.672	0.322	0.156
Day 1 Coherence	0.770	0.154	0.275
Day 1 Process Satisfaction	0.462	0.128	0.445
Day 1 Flexibility	0.848	0.678	0.823
Day 1 Process Effectiveness	0.605	0.297	0.631
Day 1 Intent to Reuse Process	0.183	0.566	0.456
Day 1 Total Completion Time	0.615	0.520	0.340

Note: Panel A reports n / mean (SD), where n is the number of participants in that row and SD is the standard deviation. DAT is Divergent Association Task. Day 1 Total Completion Time is in minutes; quality and process ratings are 7-point measures; Intent to Reuse Process is binary. Panel B1 reports each omnibus analysis of variance (ANOVA) twice: row-specific columns use all participants with that row's variable, so N can differ by row; same-256 columns use only participants with all fourteen baseline variables in Table S0a. Panel B2 reports unadjusted pairwise Welch test p-values using row-specific samples. No omnibus ANOVA survives Bonferroni correction ($\alpha = .05/14 = .00357$); the smallest same-256 result is Day 1 Interestingness ($F = 3.47$, $p = .033$).

Table S0c. Joint Tests of Baseline Balance

Variables included in joint test	N	Group sample sizes	χ^2	df	p
Four participant characteristics	280	97; 92; 91	12.67	8	0.124
Four participant characteristics plus ten Day 1 outcomes	256	91; 80; 85	28.97	28	0.414

Note: Group sample sizes are ordered as Human Creativity, Human Confirmation, and Copilot. DAT denotes Divergent Association Task. The table reports likelihood ratio tests from multinomial logistic regression models predicting randomized group assignment from baseline variables. The first row uses the four participant characteristics measured before Day 2: Creative Writing Experience, Creative Writing Ability, English Language Proficiency, and DAT Score. The second row adds the ten Day 1 baseline outcomes: Overall Quality, Originality, Interestingness, Writing Quality, Coherence, Process Satisfaction, Flexibility, Process Effectiveness, Intent to Reuse Process, and Day 1 Total Completion Time. N is the joint test sample size. χ^2 is the likelihood ratio test statistic, df denotes degrees of freedom, and p is the corresponding p-value.

Table S1. Opportunities in Large Language Model Support for Story Writing

Rank	Topics (number of reflections)	Example participant reflection
1	Idea Generation and Creativity Support (212)	"AI helped the most with ideation and outline. ChatGPT came up with ten completely different (science) fictions with twisted plots beyond the scope of everyday events."
2	Structure and Outline Assistance (180)	"By using ChatGPT to help with the outline, I was able to quickly create a structured narrative framework that guided the rest of my writing process."
3	Efficiency and Time-Saving (158)	"ChatGPT is fast at writing and better at maintaining structure than I am. It was able to follow my outline very closely and write something that had a clear beginning, conflict, and ending. When I wrote on my own, I ran out of time due to poor planning."
4	Faster Iteration and Refinement (158)	"Unsurprisingly, writing the first draft of the story with ChatGPT was much faster compared to the Day 1 assignment (10 minutes with ChatGPT vs. 64 minutes on Day 1!)."
5	Flexibility in Story Adaptation (137)	"I wanted to shift the focus of the story from one character to another midway through the narrative. ChatGPT quickly adapted by reorienting the plot and providing additional backstory for the new main character, seamlessly integrating the change into the story."
6	Diverse Perspectives and Ideas (108)	"AI can generate a list of ideas quickly, aiding in the brainstorming process. The iterative process with AI helps refine the writing to meet specific preferences."
7	Grammar and Language Improvement (91)	"I noticed that the syntax and word choice for Day 2 were far more diverse and interesting than my own writing on Day 1."
8	Enhanced Vocabulary and Description (82)	"ChatGPT provided vivid descriptions and enhanced the imagery in my story that I wouldn't have been able to come up with on my own."
9	Overcoming Writer's Block (52)	"When writing my first story without AI help, I hit some writer's blocks where ideas stopped coming for a bit. This stalled my progress. With AI helping on the second story, I flowed better as I didn't get stuck like before. Having the AI bounce ideas made it easier to just keep writing. I felt less alone when creating, less likely to bog down or lose steam."
10	Assistance with Research and Factual Accuracy (15)	"AI's vast knowledge base was useful for fact-checking and adding authenticity to story elements."

Note: We analyzed the reflections from 270 participants (Table S0a) following the method of Ludan et al. (16). After the experiment, we used large language models to identify ten positive topics across the full set and Claude Sonnet 4.6 to classify each reflection into those topics. A reflection could be assigned to none, one, or multiple topics, so the counts in parentheses need not sum to 270. Topics are ordered by frequency.

Table S2. Challenges in Large Language Model Support for Story Writing

Rank	Topics (number of reflections)	Example participant reflection
1	Difficulty in Following Complex Instructions (148)	"I gave ChatGPT detailed instructions to weave two plotlines together with alternating perspectives, but the AI struggled to maintain the structure. It either focused too much on one plotline or lost the thread of the other, leading to an unbalanced narrative."
2	Lack of Human Touch, Emotional Depth, and Authenticity (138)	"The AI's responses sometimes felt too formal and lacked the emotional depth that human storytelling often encapsulates."
3	Difficulty with Specific Editing Needs (124)	"Editing with ChatGPT was incredibly frustrating. If I prompted ChatGPT to make the story longer, the result was complex and flowery language that was unnatural to read. I also could not get ChatGPT to edit specific paragraphs or remove certain sentences."
4	Difficulty with Nuance and Context in Writing (119)	"The need for human oversight was evident; while AIs are powerful, they cannot fully grasp subjective or highly contextualized aspects."
5	Difficulty in Matching Personal Writing Style (108)	"AI's writing didn't sound like me at all; it was hard to achieve a specific writing style despite testing out various descriptions."
6	Difficulty Maintaining Logical Flow (108)	"ChatGPT sometimes generated plot points that didn't make logical sense in the context of the story. For instance, it introduced a sudden character motivation that contradicted earlier events, which disrupted the story's logical flow."
7	Overreliance on Clichés and Predictable Content (66)	"ChatGPT's writing is rather predictable. Even with additional prompting, it was pretty easy to identify which stories were written by ChatGPT."
8	Difficulty Generating Novel Ideas (57)	"Basically, quite good at creating a wide variety within one genre but not actual novel ideas"
9	Difficulty Maintaining Consistent Narrative Voice (19)	"I found that when I asked ChatGPT to continue the story from where I left off, the narrative voice often shifted noticeably. The tone and style would change, making the continuation feel disjointed from the earlier parts I had written."
10	Difficulty with Subtle Humor or Irony (6)	"When I tried to incorporate irony into the dialogue, ChatGPT often missed the mark. It would either take the statement too literally or rephrase it in a way that lost the ironic twist, making the humor fall flat."

Note: We analyzed the reflections from 270 participants (Table S0a) following the method of Ludan et al. (16). After the experiment, we used large language models to identify ten negative topics across the full set and Claude Sonnet 4.6 to classify each reflection into those topics. A reflection could be assigned to none, one, or multiple topics, so the counts in parentheses need not sum to 270. Topics are ordered by frequency.

Table S3. Changes in Total Completion Time from Day 1 to Day 2 and Contrasts Across Collaboration Designs

Panel A. Day 2 minus Day 1 changes in Total Completion Time from a model with participant fixed effects

Coefficient or contrast	Estimate	Standard error	p-value
Coefficients from the model with participant fixed effects			
Human Creativity Day 2 minus Day 1 change	-25.083	4.045	$< .001$
Human Confirmation minus Human Creativity change from Day 1 to Day 2	-0.395	5.527	0.943
Copilot minus Human Creativity change from Day 1 to Day 2	2.853	5.138	0.579
Additional Day 2 minus Day 1 changes and pairwise contrasts			
Human Confirmation Day 2 minus Day 1 change	-25.478	3.767	$< .001$
Copilot Day 2 minus Day 1 change	-22.231	3.169	$< .001$
Human Confirmation minus Copilot change from Day 1 to Day 2	-3.247	4.923	0.510

Panel B. Paired Day 2 minus Day 1 changes in Total Completion Time within each collaboration design

Collaboration design	Matched participants	Day 1 mean	Day 2 mean	Day 2 minus Day 1 mean difference	p-value from paired t-test	Cohen's d
Human Creativity	84	69.6	44.5	-25.08	$< .001$	-0.959
Human Confirmation	68	68.5	43.1	-25.48	$< .001$	-1.160
Copilot	78	68.1	45.8	-22.23	$< .001$	-1.125

Panel C. Day 1 to Day 2 changes in Total Completion Time from a model with participant characteristics as controls

Coefficient or contrast	Estimate	Standard error	p-value
Human Confirmation Day 2 minus Day 1 change	-26.420	3.678	$< .001$
Human Creativity minus Human Confirmation change from Day 1 to Day 2	0.183	5.031	0.971
Copilot minus Human Confirmation change from Day 1 to Day 2	3.299	5.115	0.519

Note: Total Completion Time is measured in minutes. Negative estimates indicate fewer minutes on Day 2 than on Day 1. Panel A estimates a difference-in-differences model with participant fixed effects, standard errors clustered by participant, and Human Creativity as the reference group. Participant fixed effects absorb time-invariant participant characteristics, so Divergent Association Task (DAT) Score, Creative Writing Experience, Creative Writing Ability, and English Language Proficiency are not separately estimated in Panel A. Panel A uses 230 matched participants and 460 participant-day observations. Panel B reports paired t-tests for the same participants. Panel C estimates a linear regression with 502 participant-day observations, Human Confirmation as the reference group, and the four participant characteristics as controls. In Panels A and C, "A minus B change from Day 1 to Day 2" means A's Day 2-minus-Day 1 change minus B's Day 2-minus-Day 1 change. Cohen's d is the standardized paired mean change, and CI denotes confidence interval. In Panel A, the Human Confirmation minus Copilot change from Day 1 to Day 2 is -3.247 minutes (95% CI: -12.90 to 6.40).

Table S4. Differences in Additional Quality Metrics Among Groups on Day 2 (Writing with AI)

Collaboration design contrast	Originality	Interestingness	Writing Quality	Coherence
Human Creativity minus Human Confirmation	-0.0409 (0.103)	0.3031*** (0.112)	0.2850*** (0.097)	0.4599*** (0.102)
Copilot minus Human Confirmation	0.0535 (0.102)	0.2614** (0.111)	0.1857* (0.097)	0.2285** (0.101)
Copilot minus Human Creativity	0.0945 (0.101)	-0.0417 (0.110)	-0.0992 (0.096)	-0.2314** (0.100)

Note: Rows are A minus B for the Day 2 quality metric in that column. Positive coefficients mean A has a higher adjusted Day 2 score; negative coefficients mean A has a lower adjusted Day 2 score. Models include controls for the four participant characteristics: Divergent Association Task (DAT) Score, Creative Writing Experience, Creative Writing Ability, and English Language Proficiency. Models are estimated at the individual evaluator-rating level. Each column reports a separate regression estimated from $N = 1{,}068$ evaluator ratings. R^2 denotes the coefficient of determination; values are .0137 (Originality), .0122 (Interestingness), .0123 (Writing Quality), and .0222 (Coherence). Outcomes are rated on a 7-point scale. Standard errors are in parentheses. Stars denote p-value thresholds: * $p < .10$, ** $p < .05$, *** $p < .01$.

Table S5. Association of Additional Quality Metrics with Overall Quality

Predictor quality metric	Overall Quality coefficient (standard error)
Constant	-0.5600*** (0.064)
Originality	0.2027*** (0.012)
Interestingness	0.4231*** (0.012)
Writing Quality	0.1671*** (0.012)
Coherence	0.2944*** (0.012)

Note: Overall Quality is the dependent variable, and Originality, Interestingness, Writing Quality, and Coherence are the predictors. Overall Quality and the predictor metrics are rated on 7-point scales. The model is estimated at the individual evaluator-rating level from $N = 2{,}196$ evaluator ratings. R^2 denotes the coefficient of determination ($R^2 = .7989$). Standard errors are in parentheses. Stars denote p-value thresholds: * $p < .10$, ** $p < .05$, *** $p < .01$.

Table S6. Completion Time Inequality on Day 2 by Day 1 Total Completion Time

Predictor	Human Confirmation		Human Creativity		Copilot	
	(1) Day 1 only	(2) Day 1 with controls	(1) Day 1 only	(2) Day 1 with controls	(1) Day 1 only	(2) Day 1 with controls
Constant	21.2859** (8.737)	21.6476 (30.632)	41.5586*** (6.199)	61.0138*** (19.617)	0.9793 (9.940)	-6.4212 (38.463)
Day 1 Total Completion Time	0.3176** (0.123)	0.3222** (0.135)	0.0426 (0.085)	-0.0049 (0.087)	0.6589*** (0.143)	0.7012*** (0.153)
DAT Score		0.1489 (0.371)		-0.1340 (0.202)		-0.1038 (0.458)
Creative Writing Experience		1.9699 (4.641)		-6.4829** (2.785)		-2.4580 (3.619)
Creative Writing Ability		0.5075 (4.047)		5.9990** (2.883)		0.4708 (4.047)
English Language Proficiency		-6.1062 (5.189)		-2.4920 (5.069)		5.9989 (5.260)

Note: Completion Time Inequality refers to persistence in participants' Total Completion Time from Day 1 to Day 2. Within each collaboration design, the table regresses Day 2 Total Completion Time, measured in minutes, on Day 1 Total Completion Time. A coefficient closer to 1 indicates stronger persistence of completion-time differences; a coefficient closer to 0 indicates flatter Day 2 times relative to Day 1. Column (1) includes Day 1 Total Completion Time only. Column (2) additionally controls for Divergent Association Task (DAT) Score, Creative Writing Experience, Creative Writing Ability, and English Language Proficiency. For columns (1) and (2), the sample sizes are 68 and 67 participants in Human Confirmation, 84 and 84 in Human Creativity, and 78 and 78 in Copilot. R^2 denotes the coefficient of determination; corresponding values are .0913 and .1133, .0030 and .0894, and .2195 and .2351. Models are estimated at the participant level. Standard errors are in parentheses. Stars denote p-value thresholds: * $p < .10$, ** $p < .05$, *** $p < .01$.

Table S7. Overall Quality by Collaboration Design on Day 1 and Day 2

Predictor	Day 1 (Without AI)		Day 2 (With AI)	
	(1) Design only	(2) Design plus controls	(1) Design only	(2) Design plus controls
Constant	4.9200*** (0.068)	3.5160*** (0.499)	4.7670*** (0.074)	5.0622*** (0.528)
**Human Creativity minus Human Confirmation**	-0.0170 (0.096)	0.0061 (0.098)	0.2780*** (0.103)	0.3376*** (0.106)
**Copilot minus Human Confirmation**	-0.1518 (0.098)	-0.1443 (0.105)	0.2628** (0.104)	0.3158*** (0.105)
DAT Score		0.0146** (0.006)		0.0007 (0.006)
Creative Writing Experience		-0.1594** (0.066)		-0.0805 (0.071)
Creative Writing Ability		0.1140 (0.070)		-0.0205 (0.074)
English Language Proficiency		0.0866 (0.097)		-0.0831 (0.101)

Note: Overall Quality is the dependent variable and is rated on a 7-point scale. Models are estimated at the individual evaluator-rating level. Human Confirmation is the reference collaboration design, so the Human Creativity and Copilot coefficients compare each design with Human Confirmation within the same day. Within each day, column (1) includes collaboration design only. Column (2) additionally controls for these participant characteristics: Divergent Association Task (DAT) Score, Creative Writing Experience, Creative Writing Ability, and English Language Proficiency. The sample sizes for columns (1) and (2), respectively, are 1,112 and 1,096 evaluator ratings on Day 1 and 1,084 and 1,068 on Day 2. R^2 denotes the coefficient of determination; corresponding values are .0026 and .0161 on Day 1 and .0084 and .0139 on Day 2. Standard errors are in parentheses. Stars denote p-value thresholds: * $p < .10$, ** $p < .05$, *** $p < .01$.

Table S8. Overall Quality Inequality on Day 2 by Day 1 Overall Quality

Predictor	Human Confirmation		Human Creativity		Copilot	
	(1) Day 1 only	(2) Day 1 with controls	(1) Day 1 only	(2) Day 1 with controls	(1) Day 1 only	(2) Day 1 with controls
Constant	4.5196*** (0.570)	3.6482*** (1.321)	4.0526*** (0.438)	5.3392*** (0.781)	4.1926*** (0.454)	4.9040*** (1.171)
**Day 1 Overall Quality**	0.0497 (0.114)	0.0864 (0.114)	0.2016** (0.088)	0.2442** (0.093)	0.1774* (0.094)	0.2069** (0.095)
DAT Score		0.0112 (0.014)		-0.0054 (0.008)		-0.0059 (0.014)
Creative Writing Experience		-0.1429 (0.155)		0.0746 (0.103)		-0.1106 (0.124)
Creative Writing Ability		-0.1974 (0.151)		-0.1255 (0.108)		0.2695** (0.133)
English Language Proficiency		0.1158 (0.183)		-0.3284* (0.184)		-0.2545 (0.173)

Note: Overall Quality Inequality refers to persistence in participants' Overall Quality from Day 1 to Day 2. Within each collaboration design, the table regresses Day 2 Overall Quality on Day 1 Overall Quality. Overall Quality is rated on a 7-point scale. A coefficient closer to 1 indicates stronger persistence of quality differences; a coefficient closer to 0 indicates flatter Day 2 quality relative to Day 1. Column (1) includes Day 1 Overall Quality only. Column (2) additionally controls for Divergent Association Task (DAT) Score, Creative Writing Experience, Creative Writing Ability, and English Language Proficiency. For columns (1) and (2), the sample sizes are 86 and 83 participants in Human Confirmation, 90 and 90 in Human Creativity, and 88 and 88 in Copilot. R^2 denotes the coefficient of determination; corresponding values are .0022 and .0748, .0568 and .1231, and .0398 and .1063. Models are estimated at the participant level. Standard errors are in parentheses. Stars denote p-value thresholds: * $p < .10$, ** $p < .05$, *** $p < .01$.

Table S9. Overall Quality Inequality by DAT Score on Day 1 and Day 2

Predictor	Day 1 (Without AI)		Day 2 (With AI)	
	(1) DAT Score	(2) DAT Score plus other participant characteristics	(1) DAT Score	(2) DAT Score plus other participant characteristics
Constant	3.6088*** (0.460)	3.5091*** (0.496)	5.0645*** (0.491)	5.2458*** (0.528)
**DAT Score**	0.0153*** (0.006)	0.0139** (0.006)	-0.0015 (0.006)	-0.0005 (0.006)
Creative Writing Experience		-0.1673** (0.066)		-0.0762 (0.071)
Creative Writing Ability		0.1144 (0.070)		-0.0049 (0.075)
English Language Proficiency		0.0961 (0.096)		-0.0469 (0.101)

Note: DAT denotes Divergent Association Task. This table reports Overall Quality inequality by regressing Overall Quality on DAT Score separately for Day 1 and Day 2. Overall Quality is rated on a 7-point scale, and models are estimated at the evaluator-rating level. Here, inequality refers to the association between DAT Score and evaluated story quality, rather than the Day 1 to Day 2 persistence reported in Tables S6 and S8. A positive coefficient indicates that participants with higher DAT Scores receive higher quality ratings; a coefficient closer to 0 indicates a flatter relationship. Both columns include DAT Score. Column (2) additionally controls for Creative Writing Experience, Creative Writing Ability, and English Language Proficiency. For columns (1) and (2), the sample sizes are 1,096 and 1,096 evaluator ratings on Day 1 and 1,068 and 1,068 on Day 2. R^2 denotes the coefficient of determination; corresponding values are .0069 and .0134 on Day 1 and .0001 and .0021 on Day 2. Standard errors are in parentheses. Stars denote p-value thresholds: * $p < .10$, ** $p < .05$, *** $p < .01$.

Table S10. Day 2 Minus Day 1 Changes in Process Satisfaction Measures by Collaboration Design

Collaboration design	Day 2 minus Day 1 change			
	Process Satisfaction	Flexibility	Process Effectiveness	Intent to Reuse Process
Human Confirmation	-0.0507 (0.248)	-0.7975** (0.242)	-0.0715 (0.266)	-0.1320* (0.074)
Human Creativity	0.8584*** (0.215)	-0.0050 (0.213)	0.8491*** (0.225)	0.1510** (0.065)
Copilot	0.4154* (0.228)	0.0484 (0.221)	0.8548*** (0.216)	0.1484** (0.067)

Note: Each cell reports the Day 2 minus Day 1 change for one collaboration design and one process satisfaction measure. Positive coefficients indicate higher values on Day 2 than on Day 1. Models include controls for Divergent Association Task (DAT) Score, Creative Writing Experience, Creative Writing Ability, and English Language Proficiency. Process Satisfaction, Flexibility, and Process Effectiveness are measured on 7-point scales. Intent to Reuse Process is binary, so its coefficient is a difference in probability. Each cell comes from a separate participant-level regression using that group's Day 1 and Day 2 reports. The samples are $N =$ 158 participant-days for Human Confirmation, 179 for Human Creativity, and 167 for Copilot. R^2 denotes the coefficient of determination; values for Human Confirmation, Human Creativity, and Copilot are .0521, .1464, and .0236 for Process Satisfaction; .0772, .0452, and .0115 for Flexibility; .0357, .1105, and .0934 for Process Effectiveness; and .0384, .0828, and .0373 for Intent to Reuse Process. Standard errors are in parentheses. Stars denote p-value thresholds: * $p < .10$, ** $p < .05$, *** $p < .01$.

Table S11. Group Differences in Process Satisfaction Measures on Day 2 (Writing with AI)

Collaboration design contrast	Process Satisfaction	Flexibility	Process Effectiveness	Intent to Reuse Process
Human Creativity minus Human Confirmation	0.6766*** (0.250)	0.7848*** (0.252)	0.8023*** (0.249)	0.1957*** (0.067)
Copilot minus Human Confirmation	0.5793** (0.251)	0.8692*** (0.253)	1.0316*** (0.251)	0.2159*** (0.067)
Copilot minus Human Creativity	-0.0972 (0.247)	0.0845 (0.248)	0.2293 (0.246)	0.0202 (0.066)

Note: Rows are A minus B for the Day 2 process satisfaction measure in that column. Positive coefficients mean A has a higher adjusted Day 2 value; negative coefficients mean A has a lower adjusted Day 2 value. Models include controls for the four participant characteristics: Divergent Association Task (DAT) Score, Creative Writing Experience, Creative Writing Ability, and English Language Proficiency. Models are estimated at the participant level. Process Satisfaction, Flexibility, and Process Effectiveness are measured on 7-point scales. Intent to Reuse Process is binary, so its coefficient is a difference in probability. Each column reports a separate regression estimated from $N = 246$ participants. R^2 denotes the coefficient of determination; values are .0579 (Process Satisfaction), .0712 (Flexibility), .0946 (Process Effectiveness), and .0842 (Intent to Reuse Process). Standard errors are in parentheses. Stars denote p-value thresholds: * $p < .10$, ** $p < .05$, *** $p < .01$.

Table S12. Group Differences in AI Effectiveness, Satisfaction with AI, and AI's Contribution on Day 2 (Writing with AI)

Collaboration design contrast	AI Effectiveness	Satisfaction with AI	AI's Contribution
Human Creativity minus Human Confirmation	0.4987** (0.225)	0.7336*** (0.251)	-20.5582*** (2.381)
Copilot minus Human Confirmation	0.5087** (0.226)	1.0134*** (0.253)	-13.5237*** (2.368)
Copilot minus Human Creativity	0.0100 (0.222)	0.2798 (0.248)	7.0345*** (2.337)

Note: The first two columns are 7-point ratings of AI Effectiveness and Satisfaction with AI. AI's Contribution is a 0–100 percentage measure of ChatGPT's contribution. Rows are A minus B. Positive coefficients mean A has a higher adjusted Day 2 rating or reported AI contribution; negative coefficients mean A has a lower adjusted Day 2 rating or reported AI contribution. Models include controls for the four participant characteristics: Divergent Association Task (DAT) Score, Creative Writing Experience, Creative Writing Ability, and English Language Proficiency. Models are estimated at the participant level. Each column reports a separate regression estimated from $N = 246$ participants. R^2 denotes the coefficient of determination; values are .0394 (AI Effectiveness), .0970 (Satisfaction with AI), and .2619 (AI's Contribution). Standard errors are in parentheses. Stars denote p-value thresholds: * $p < .10$, ** $p < .05$, *** $p < .01$.

Table S13. Potential Human-AI Collaboration Designs in the Creative Process

Table S13 organizes writing designs by who leads pre-production (ideation and outlining) and who leads production (drafting and editing). The empirical analyses focus on three human-AI collaboration designs: Human Creativity, Human Confirmation, and Copilot.

	**Production lead**	
**Pre-production lead**	**Human leads production**	**AI leads production**
**Human leads pre-production**	Human Only	Human Creativity
**AI leads pre-production**	AI Creativity	Human Confirmation

Note: Pre-production refers to ideation and outlining; production refers to drafting and editing. Human Only denotes the Day 1 baseline without AI. Human Creativity, Human Confirmation, and Copilot are the three Day 2 collaboration designs studied in the experiment. AI Creativity denotes a possible design that was not studied. Copilot is not shown as a single matrix cell because participants could use AI assistance throughout both phases while retaining control over the writing process.

Table S14. Changes in Story Quality from Day 1 to Day 2 and Contrasts Across Collaboration Designs

Panel A. Changes in story quality from Day 1 to Day 2 from models with participant fixed effects

Dimension	Estimate reported	Evaluator rating estimate (SE)	*p*-value	Participant-day estimate (SE)	*p*-value
Overall Quality	Human Creativity Day 2 minus Day 1 change	0.106 (0.097)	0.276	0.106 (0.131)	0.420
Overall Quality	Human Confirmation change minus Human Creativity change from Day 1 to Day 2	-0.271 (0.154)	0.079	-0.271 (0.208)	0.194
Overall Quality	Copilot change minus Human Creativity change from Day 1 to Day 2	0.162 (0.144)	0.259	0.162 (0.194)	0.404
Originality	Human Creativity Day 2 minus Day 1 change	-0.006 (0.105)	0.958	-0.006 (0.142)	0.969
Originality	Human Confirmation change minus Human Creativity change from Day 1 to Day 2	0.136 (0.155)	0.378	0.136 (0.209)	0.514
Originality	Copilot change minus Human Creativity change from Day 1 to Day 2	0.273 (0.157)	0.083	0.273 (0.212)	0.199
Interestingness	Human Creativity Day 2 minus Day 1 change	0.025 (0.099)	0.802	0.025 (0.134)	0.852
Interestingness	Human Confirmation change minus Human Creativity change from Day 1 to Day 2	-0.266 (0.161)	0.099	-0.266 (0.218)	0.222
Interestingness	Copilot change minus Human Creativity change from Day 1 to Day 2	0.214 (0.149)	0.150	0.214 (0.201)	0.287
Writing Quality	Human Creativity Day 2 minus Day 1 change	0.564 (0.114)	$<.001$	0.564 (0.153)	$<.001$
Writing Quality	Human Confirmation change minus Human Creativity change from Day 1 to Day 2	-0.302 (0.164)	0.066	-0.302 (0.222)	0.174
Writing Quality	Copilot change minus Human Creativity change from Day 1 to Day 2	0.050 (0.164)	0.762	0.050 (0.222)	0.823
Coherence	Human Creativity Day 2 minus Day 1 change	0.272 (0.098)	0.005	0.272 (0.132)	0.040
Coherence	Human Confirmation change minus Human Creativity change from Day 1 to Day 2	-0.426 (0.150)	0.004	-0.426 (0.202)	0.035
Coherence	Copilot change minus Human Creativity change from Day 1 to Day 2	-0.048 (0.143)	0.737	-0.048 (0.193)	0.804

Panel B. Contrasts across collaboration designs in Day 2 minus Day 1 changes in story quality from models with participant fixed effects

Dimension	Pairwise change contrast	Evaluator rating estimate (SE)	*p*-value	Participant-day estimate (SE)	*p*-value
Overall Quality	Human Confirmation change minus Human Creativity change from Day 1 to Day 2	-0.271 (0.154)	0.079	-0.271 (0.208)	0.194
Overall Quality	Copilot change minus Human Creativity change from Day 1 to Day 2	0.162 (0.144)	0.259	0.162 (0.194)	0.404
Overall Quality	Human Confirmation change minus Copilot change from Day 1 to Day 2	-0.433 (0.160)	0.007	-0.433 (0.216)	0.046
Originality	Human Confirmation change minus Human Creativity change from Day 1 to Day 2	0.136 (0.155)	0.378	0.136 (0.209)	0.514
Originality	Copilot change minus Human Creativity change from Day 1 to Day 2	0.273 (0.157)	0.083	0.273 (0.212)	0.199
Originality	Human Confirmation change minus Copilot change from Day 1 to Day 2	-0.136 (0.163)	0.404	-0.136 (0.220)	0.537
Interestingness	Human Confirmation change minus Human Creativity change from Day 1 to Day 2	-0.266 (0.161)	0.099	-0.266 (0.218)	0.222
Interestingness	Copilot change minus Human Creativity change from Day 1 to Day 2	0.214 (0.149)	0.150	0.214 (0.201)	0.287
Interestingness	Human Confirmation change minus Copilot change from Day 1 to Day 2	-0.480 (0.168)	0.004	-0.480 (0.228)	0.035
Writing Quality	Human Confirmation change minus Human Creativity change from Day 1 to Day 2	-0.302 (0.164)	0.066	-0.302 (0.222)	0.174
Writing Quality	Copilot change minus Human Creativity change from Day 1 to Day 2	0.050 (0.164)	0.762	0.050 (0.222)	0.823
Writing Quality	Human Confirmation change minus Copilot change from Day 1 to Day 2	-0.352 (0.168)	0.036	-0.352 (0.227)	0.121
Coherence	Human Confirmation change minus Human Creativity change from Day 1 to Day 2	-0.426 (0.150)	0.004	-0.426 (0.202)	0.035

Table S14, Panel B continued

Dimension	Pairwise change contrast	Evaluator rating estimate (SE)	p-value	Participant-day estimate (SE)	p-value
Coherence	Copilot change minus Human Creativity change from Day 1 to Day 2	-0.048 (0.143)	0.737	-0.048 (0.193)	0.804
Coherence	Human Confirmation change minus Copilot change from Day 1 to Day 2	-0.379 (0.153)	0.014	-0.379 (0.207)	0.068

Note: Table S14 reports changes in story quality from Day 1 (writing without AI) to Day 2 (writing with AI). Story quality outcomes are rated on 7-point scales. Panel A uses Human Creativity as the reference collaboration design. It reports the Human Creativity Day 2-minus-Day 1 change and then asks whether the Human Confirmation and Copilot changes differ from the Human Creativity change. Panel B reports all pairwise comparisons among the three collaboration designs. In Panel B, "A minus B" means A's Day 2-minus-Day 1 change minus B's Day 2-minus-Day 1 change; a negative coefficient means A's change is lower than B's change. Both panels report two versions of the same participant fixed-effects difference-in-differences model. The evaluator-rating model uses 2,196 evaluator ratings over 549 participant-day stories from 285 participants and clusters standard errors by participant. The participant-day model first averages evaluator ratings within each story and then estimates the same model from 549 participant-day means, also clustering standard errors by participant. Point estimates match across the two versions, but standard errors differ because the participant-day version averages raters before estimation. Estimates are regression coefficients, SE denotes standard error, and p-values test whether the coefficient differs from 0. Participant fixed effects absorb time-invariant participant characteristics, so Divergent Association Task (DAT) Score, Creative Writing Experience, Creative Writing Ability, and English Language Proficiency are not separately estimated in Table S14. Table S15 reports the matched quality sample used for paired tests. Tables S4 and S7 report Day 2 level differences with controls for participant characteristics, not difference-in-differences estimates.

Table S15. Paired Day 2 Minus Day 1 Changes in Story Quality

Collaboration design	Quality dimension	N	Day 1 mean	Day 2 mean	Day 2 minus Day 1	Paired test p-value	Cohen's d
Human Creativity	Overall Quality	90	4.94	5.05	0.106	0.248	0.123
Human Creativity	Originality	90	4.96	4.96	-0.006	0.955	-0.006
Human Creativity	Interestingness	90	4.94	4.96	0.025	0.789	0.028
Human Creativity	Writing Quality	90	5.03	5.59	0.564	$< .001$	0.559
Human Creativity	Coherence	90	5.23	5.50	0.272	0.004	0.313
Human Confirmation	Overall Quality	86	4.93	4.76	-0.165	0.146	-0.158
Human Confirmation	Originality	86	4.93	5.06	0.131	0.222	0.133
Human Confirmation	Interestingness	86	4.96	4.72	-0.241	0.046	-0.218
Human Confirmation	Writing Quality	86	5.04	5.30	0.262	0.021	0.254
Human Confirmation	Coherence	86	5.19	5.04	-0.154	0.149	-0.157
Copilot	Overall Quality	88	4.77	5.04	0.268	0.008	0.287
Copilot	Originality	88	4.82	5.09	0.267	0.017	0.260
Copilot	Interestingness	88	4.70	4.94	0.239	0.023	0.246
Copilot	Writing Quality	88	4.87	5.49	0.614	$< .001$	0.589
Copilot	Coherence	88	5.04	5.26	0.224	0.023	0.247

Note: Tests are paired within participant using participant-day quality scores averaged across raters. Story quality outcomes are rated on a 7-point scale. N is the number of participants with quality scores on both days. The Day 2 minus Day 1 column reports the mean difference within the same participants. The paired test p-value is from a paired t-test, and Cohen's d is the standardized paired mean change. Negative values indicate lower quality on Day 2 than on Day 1.

Table S16. Paired Day 2 Minus Day 1 Changes in Process Satisfaction Measures

Collaboration design	Process Satisfaction measure	N	Day 1 mean	Day 2 mean	Day 2 minus Day 1	Paired test p-value	Cohen's d
Human Creativity	Process Satisfaction	84	4.54	5.31	0.774	0.001	0.362
Human Creativity	Flexibility	84	5.04	4.96	-0.071	0.746	-0.035
Human Creativity	Process Effectiveness	84	4.36	5.13	0.774	$< .001$	0.383
Human Creativity	Intent to Reuse Process	84	0.65	0.79	0.131	0.048	0.219
Human Confirmation	Process Satisfaction	70	4.79	4.69	-0.100	0.686	-0.049
Human Confirmation	Flexibility	70	5.10	4.31	-0.786	0.002	-0.392
Human Confirmation	Process Effectiveness	70	4.54	4.44	-0.100	0.721	-0.043
Human Confirmation	Intent to Reuse Process	70	0.76	0.63	-0.129	0.118	-0.189
Copilot	Process Satisfaction	78	4.82	5.27	0.449	0.071	0.207
Copilot	Flexibility	78	5.10	5.14	0.038	0.875	0.018
Copilot	Process Effectiveness	78	4.55	5.38	0.833	0.001	0.384
Copilot	Intent to Reuse Process	78	0.65	0.82	0.167	0.027	0.255

Note: Tests are paired within participant using the matched Day 1 and Day 2 process satisfaction sample. N is the number of participants with process satisfaction reports on both days. Process Satisfaction, Flexibility, and Process Effectiveness are 7-point measures. Intent to Reuse Process is binary, so its means and changes are proportions. Columns "Day 1 mean" and "Day 2 mean" report group means. The Day 2 minus Day 1 column reports the mean difference within the same participants. The paired test p-value is from a paired t-test, and Cohen's d is the standardized paired mean change.

Table S17. Pairwise Contrasts in Day 2 Minus Day 1 Changes in Process Satisfaction Measures

Process satisfaction measure	Collaboration-design contrast	Estimate (standard error)	p-value
Process Satisfaction	Human Confirmation minus Human Creativity	-0.874 (0.480)	0.069
Process Satisfaction	Human Creativity minus Copilot	0.325 (0.479)	0.497
Process Satisfaction	Human Confirmation minus Copilot	-0.549 (0.492)	0.264
Flexibility	Human Confirmation minus Human Creativity	-0.714 (0.461)	0.121
Flexibility	Human Creativity minus Copilot	-0.110 (0.464)	0.813
Flexibility	Human Confirmation minus Copilot	-0.824 (0.483)	0.088
Process Effectiveness	Human Confirmation minus Human Creativity	-0.874 (0.503)	0.082
Process Effectiveness	Human Creativity minus Copilot	-0.060 (0.468)	0.899
Process Effectiveness	Human Confirmation minus Copilot	-0.933 (0.526)	0.076
Intent to Reuse Process	Human Confirmation minus Human Creativity	-0.260 (0.147)	0.078
Intent to Reuse Process	Human Creativity minus Copilot	-0.036 (0.140)	0.798
Intent to Reuse Process	Human Confirmation minus Copilot	-0.295 (0.155)	0.057

Note: Each row reports a pairwise contrast in Day 2 minus Day 1 changes from a model with participant fixed effects and two waves of data. In the contrast column, "A minus B" means A's Day 2-minus-Day 1 change minus B's Day 2-minus-Day 1 change. Process Satisfaction, Flexibility, and Process Effectiveness are 7-point measures. Intent to Reuse Process is binary, so its coefficients are differences in probability. The matched sample has 232 participants and 464 participant-day observations. Estimates are regression coefficients, standard errors are in parentheses, and p-values test whether the coefficient differs from 0. Negative estimates mean A has a lower Day 2-minus-Day 1 change than B. Participant fixed effects absorb time-invariant participant characteristics. Table S10 reports the within-design Day 2 minus Day 1 changes; Table S11 reports Day 2 group comparisons for the same process satisfaction measures; Table S12 reports Day 2 group comparisons for AI Effectiveness, Satisfaction with AI, and AI's Contribution.

Table S18. Genre Classification of Story Records Using GPT-4o and Qwen2.5-72B-Instruct

Panel A. Agreement between GPT-4o and Qwen genre classifications

Sample count or agreement statistic	Value
Story records used in the genre analysis	557
Day 1 records	282
Day 2 Human Creativity records	93
Day 2 Human Confirmation records	91
Day 2 Copilot records	91
Qwen priority labels outside the nine-genre scheme	5 of 557
Qwen records with any label outside the nine-genre scheme	108 of 557
Priority genre exact agreement	76.5% (95% bootstrap CI: 72.9%, 79.9%)
Priority genre unweighted Cohen's κ	0.719 (95% bootstrap CI: 0.677, 0.761)
Mean Jaccard overlap for top three genre labels	0.720
Micro-averaged F1 score across nine binary genre labels	0.791
Macro-averaged F1 score across nine binary genre labels	0.745
Hamming agreement across nine binary genre labels	84.3%
Pearson correlation across all condition and genre cells	0.902 (36 cells; 95% CI: 0.816, 0.949)
Spearman correlation across all condition and genre cells	0.873 (36 cells)
Pearson correlation across Day 2 condition and genre cells	0.904 (27 cells; 95% CI: 0.799, 0.956)
Spearman correlation across Day 2 condition and genre cells	0.874 (27 cells)
Mean absolute GPT-4o versus Qwen prevalence difference	8.2 pp
Largest absolute GPT-4o versus Qwen prevalence difference	31.2 pp

Panel B. Genre measures by classification model

Condition	Classification model	Story records	Mean labels per record	Mean cosine similarity	Any plot-driven genre (%)	Any personal or experiential genre (%)
Writing without AI	GPT-4o	282	3.447	0.532	84.0	89.4
Writing without AI	Qwen	282	2.915	0.471	88.7	73.8
Human Creativity	GPT-4o	93	3.548	0.533	92.5	91.4
Human Creativity	Qwen	93	3.097	0.476	94.6	65.6
Human Confirmation	GPT-4o	91	4.066	0.592	97.8	64.8
Human Confirmation	Qwen	91	3.648	0.597	98.9	47.3
Copilot	GPT-4o	91	3.758	0.527	94.5	72.5
Copilot	Qwen	91	3.352	0.504	96.7	54.9

Note: Table S18 compares post-experiment genre classifications by GPT-4o (17) and Qwen2.5-72B-Instruct (18). These classifiers are separate from the AI assistance that participants used while writing. The comparison supports the broad group-level genre pattern rather than validating the individual genre labels. Both classifiers place Human Confirmation highest in mean labels per story and in within-condition genre similarity, but Qwen assigns some stories labels outside the nine-genre scheme, macro-F1 is below one, and prevalence differs substantially for some genres. The analysis uses $N = 557$ story records: Day 1 $n = 282$, Day 2 Human Creativity $n = 93$, Human Confirmation $n = 91$, and Copilot $n = 91$. This record set includes eight records outside the 549 story records used in the quality analyses; Figure S0 and Table S0a show how stories enter those analyses.

Both classifiers use the same nine multi-label genres: Horror Fiction, Mystery/Crime Fiction, Romance, Sci-Fi, Thriller/Suspense, Western Fiction, Historical Fiction, Young Adult Fiction, and Fantasy. Percentages can exceed 100 because one story record can receive multiple genre labels.

A priority label is the primary genre label assigned to a record. The top three genre labels are the three highest-priority labels. Priority agreement and Cohen's κ treat Qwen priority labels outside the nine-genre scheme as a separate category. Qwen labels outside the scheme are dropped before constructing the nine binary genre indicators.

Jaccard overlap compares the two classifiers' top-three label sets. Micro-averaged F1, macro-averaged F1, and Hamming agreement are computed from the nine binary genre indicators.

Prevalence correlations compare GPT-4o and Qwen percentages across condition-by-genre cells. Prevalence differences are absolute GPT-4o versus Qwen differences in percentage points; the largest is Young Adult Fiction in Day 2 Human Creativity (74.2% with GPT-4o and 43.0% with Qwen). Mean cosine similarity uses the nine genre labels within each condition; records with no in-scheme genres are retained and zero-vector pairs are coded as 0.

Plot-driven genres are Fantasy, Sci-Fi, Mystery/Crime Fiction, and Thriller/Suspense. Personal or experiential genres are Young Adult Fiction, Historical Fiction, and Horror Fiction. Romance and Western Fiction are not included in either set. CI denotes a 95% confidence interval, and pp denotes percentage points.

Table S19. Coding Definitions for the Reflection-Essay Analysis

Process indicators (who did each step)	
Initial idea (AI / human)	whether the AI, or the participant, produced the initial story idea.
Outline (AI / human)	whether the AI, or the participant, produced the outline or structure.
AI drafted core story	the AI wrote the main prose or first full draft.
Human drafted / rewrote	the participant wrote prose or substantially rewrote the AI output.
Iterative prompting	back-and-forth prompting or repeated refinement of the output.
Human final editing	the participant reviewed, selected, or did final editing of the output.
Experience themes (rated 0–3)	
Expressed pride	pride in, or a sense of having authored, the final story.
Expressed partnership	feeling like a genuine collaborator with the AI, rather than an editor or bystander.
Loss of authorship	feeling the output was not theirs.
Loss of creative agency	being reduced to approving the AI's choices.
Suppression of personal voice	the AI failing to carry the participant's own voice or style.
Coherence problem	coherence, structure, or nuance problems in the AI output.
Other themes	passivity, effort reduction, idea stimulation (reported in Table S21).

Note: Coding definitions for the analysis of the 270 reflection essays. Process indicators are binary, coded present only when the participant explicitly stated it. Experience themes are rated from 0 (absent) to 3 (strong) in intensity.

Table S20. Process Coding of the Reflection Essays

Process indicator	HC	HCr	CO	p	Pairwise
**AI generated initial idea**	**80.2**	**8.5**	**57.6**	$< .001$	HC > CO > HCr
**Human generated initial idea**	**2.2**	**77.7**	**40.0**	$< .001$	HCr > CO > HC
**AI created outline**	**54.9**	**6.4**	**49.4**	$< .001$	HC ≈ CO; HCr lowest
**Human created outline**	**13.2**	**77.7**	**22.4**	$< .001$	HCr highest; HC ≈ CO
AI drafted core story	87.9	94.7	90.6	.265	no difference
Human drafted / rewrote	12.1	13.8	28.2	.009	CO highest
Iterative prompting	91.2	92.6	97.6	.182	no difference
Human final editing	51.6	58.5	69.4	.054	CO highest

Note: Process coding of the 270 reflection essays ($n = 270$; HC 91, HCr 94, CO 85). Each entry is the percentage of essays in that design showing the indicator; p is from a chi-square test across the three designs. Pairwise patterns are descriptive. Bold rows mark the early idea and outline stages, where the designs differ most. "No difference" denotes $p \geq .05$. The remaining coded process problems (difficulty steering, voice or style mismatch, coherence, and ownership or distance) showed no significant design differences except coherence (discussed in the text) and are omitted here for concision. HC, Human Confirmation; HCr, Human Creativity; CO, Copilot.

Table S21. Experience Coding of the Reflection Essays

Theme	HC	HCr	CO	p
**Expressed partnership**	**0.84**	**1.02**	**1.18**	**.0025**
Expressed pride	0.24	0.54	0.40	.0052
Coherence problem	1.32	1.04	1.22	.040
Effort reduction	1.30	1.55	1.45	.042
Process enjoyment	0.66	0.79	0.87	.089 (ns)
Passivity	0.22	0.11	0.13	.088 (ns)
Idea stimulation	0.95	0.60	1.05	.125 (ns)†
Loss of creative agency	0.73	0.64	0.54	.270 (ns)
Loss of authorship	0.25	0.24	0.25	.647 (ns)
Suppression of personal voice	0.84	0.83	0.76	.740 (ns)

Note: Experience coding, mean intensity (0–3) for the 270 reflection essays (HC 91, HCr 94, CO 85). The p value is from a Mann–Whitney test of Human Confirmation versus the two human-led designs combined (Human Creativity and Copilot). All ten coded themes are shown. The bold row survives a Bonferroni correction ($\alpha = .005$). Unbolded rows with $p < .05$ are nominal only; rows marked "ns" have $p \geq .05$. †Idea stimulation does not differ for that contrast, but differs between the two human-led designs (higher under Copilot, $p < .001$). HC, Human Confirmation; HCr, Human Creativity; CO, Copilot.

Table S22. Validation of the Automated Coding Against a Human Coder

To assess the reliability of the automated coding of the reflection essays, a human coder re-coded a stratified random 20% subsample (54 of the 270 essays; 18 Human Confirmation, 19 Human Creativity, 17 Copilot), blind to the automated labels and using the same coding rubric. Before coding the subsample, the coder completed a calibration pass on ten essays not included in it. Table S22 reports agreement between the human and automated codings for each code; overall figures pool all coded decisions rather than averaging the per-code percentages.

The eight process indicators are binary. The ten experience themes are rated on a four-point intensity scale, the scale compared in Table S21; for these we report agreement within one scale point and exact agreement.

Agreement on the process indicators was 87.5% overall and 83–93% for the codes that identify who generated the initial idea and outline. It was lower for the two broader codes (human drafting or rewriting, 85%; final editing, 70%), which cover overlapping actions. For the experience themes, the two codings agreed within one scale point on 87% of ratings and exactly on 48%; for expressed pride and expressed partnership, the themes that bear on the reported differences, within-one agreement was 93–100%.

Code	Within one level (%)	Exact (%)
Process indicators (binary)		
AI generated the initial idea	—	83
Human generated the initial idea	—	89
AI created the outline	—	89
Human created the outline	—	93
AI drafted the core story	—	96
Human drafted or majorly rewrote	—	85
Iterative prompting	—	94
Human final editing	—	70
**Overall**	—	**87.5**
Experience themes (0–3 intensity)		
Expressed partnership	100	48
Expressed pride	93	67
Coherence problem	85	33
Effort reduction	96	50
Process enjoyment	93	48
Passivity	78	54
Idea stimulation	70	26
Loss of creative agency	87	44
Loss of authorship	87	69
Suppression of personal voice	78	39
**Overall**	**87**	**48**

Note: Agreement between the human coder and the automated coding on a stratified random 20% subsample of the reflection essays ($n = 54$). Process indicators are binary and are compared directly, so only exact agreement applies. Experience themes are rated on a four-point intensity scale (0, absent; 3, strong), the scale compared in Table S21; "within one level" counts ratings that differ by at most one point, and "exact" counts identical ratings. Overall figures pool all coded decisions. We report percent agreement because several process indicators have highly skewed base rates (for example, iterative prompting appears in over 90% of essays in every design; Table S20), where chance-corrected coefficients are unstable and understate agreement. Theme order matches Table S21.

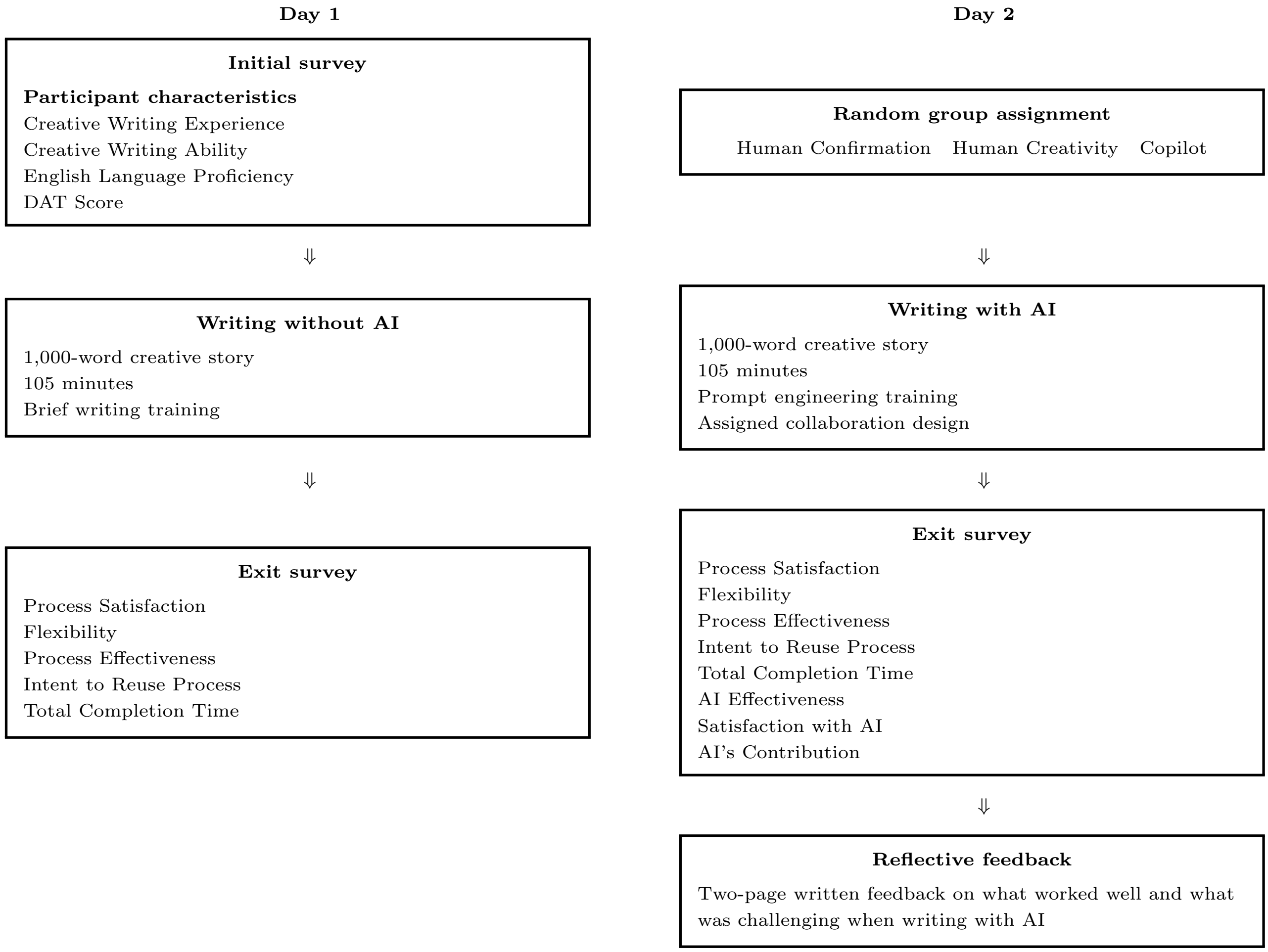

Figure S1. Experimental design. The study used a two-day within-participant protocol. On Day 1, all participants wrote a 1,000-word story without AI. On Day 2, each participant was randomly assigned to one of three human–AI collaboration designs. In Human Creativity, participants created the idea and outline while ChatGPT drafted and edited the story. In Human Confirmation, ChatGPT generated the idea, outline, draft, and edits while participants prompted and confirmed or rejected outputs. In Copilot, participants could use ChatGPT throughout ideation, outlining, drafting, and editing while retaining control over the writing process. DAT denotes Divergent Association Task.

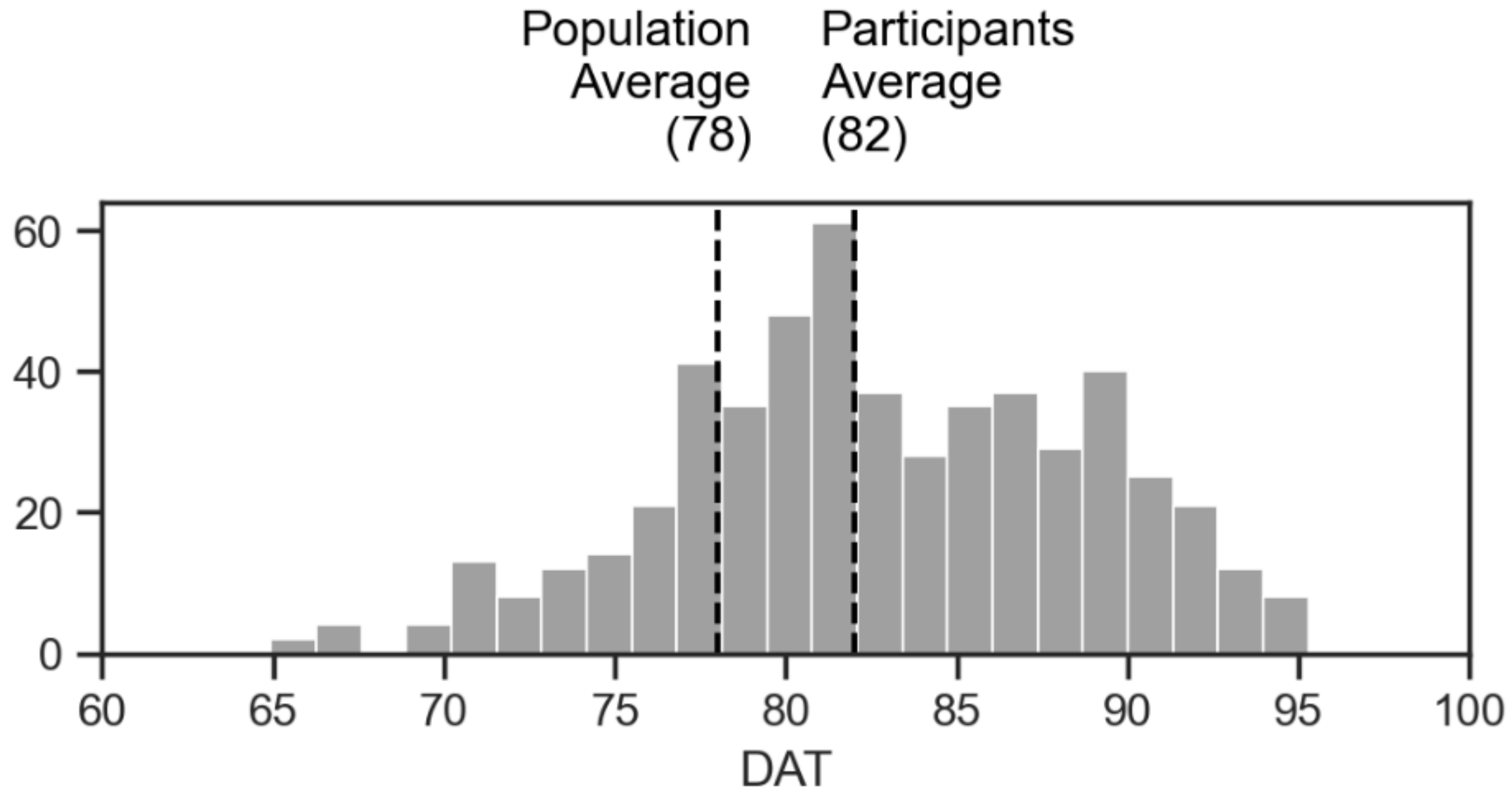

Figure S2. Distribution of Divergent Association Task (DAT) scores for the 280 participants who completed the DAT measure. The score measures verbal creativity from the semantic distance among ten participant-generated nouns; higher scores indicate more conceptually distant word sets. The cohort mean is 82.28.

SI References

1. T. I. Lubart, Models of the creative process: Past, present and future. *Creativity research journal* **13**, 295–308 (2001).
2. D. Callele, E. Neufeld, K. Schneider, Requirements engineering and the creative process in the video game industry. In *Proceedings of the 13th IEEE International Conference on Requirements Engineering* (IEEE, 2005), pp. 240–250.
3. J. Hitsuwari, Y. Ueda, W. Yun, M. Nomura, Does human–AI collaboration lead to more creative art? Aesthetic evaluation of human-made and AI-generated haiku poetry. *Computers in Human Behavior* **139**, 107502 (2023).
4. N. Köbis, L. D. Mossink, Artificial intelligence versus Maya Angelou: Experimental evidence that people cannot differentiate AI-generated from human-written poetry. *Computers in human behavior* **114**, 106553 (2021).
5. J. Nielsen, Ideation Is Free: AI Exhibits Strong Creativity, But AI-Human Co-Creation Is Better. *Jakob Nielsen on UX* (2023). Available at: https://jakobnielsenphd.substack.com/p/ideation-is-free-ai-strong-creativity [Accessed 28 August 2024].
6. N. Jia, X. Luo, Z. Fang, C. Liao, When and how artificial intelligence augments employee creativity. *Academy of Management Journal* **67**, 5–32 (2024).
7. S. Peng, E. Kalliamvakou, P. Cihon, M. Demirer, The impact of ai on developer productivity: Evidence from github copilot. *arXiv preprint arXiv:2302.06590* (2023).
8. J. A. Olson, J. Nahas, D. Chmoulevitch, S. J. Cropper, M. E. Webb, Naming unrelated words predicts creativity. *Proceedings of the National Academy of Sciences* **118**, e2022340118 (2021).
9. D. Cropley, Is artificial intelligence more creative than humans?: ChatGPT and the divergent association task. *Learning Letters* **2**, 13–13 (2023).
10. A. R. Doshi, O. P. Hauser, Generative AI enhances individual creativity but reduces the collective diversity of novel content. *Science Advances* **10**, eadn5290 (2024).
11. K. F. Hubert, K. N. Awa, D. L. Zabelina, The current state of artificial intelligence generative language models is more creative than humans on divergent thinking tasks. *Scientific Reports* **14**, 3440 (2024).
12. R. D'Souza, What characterises creativity in narrative writing, and how do we assess it? Research findings from a systematic literature search. *Thinking skills and creativity* **42**, 100949 (2021).
13. A. R. Fabbri, *et al.*, Summeval: Re-evaluating summarization evaluation. *Transactions of the Association for Computational Linguistics* **9**, 391–409 (2021).
14. M. Adam, K. Roethke, A. Benlian, Human vs. automated sales agents: How and why customer responses shift across sales stages. *Information Systems Research* **34**, 1148–1168 (2023).
15. S. Noy, W. Zhang, Experimental evidence on the productivity effects of generative artificial intelligence. *Science* **381**, 187–192 (2023).
16. J. M. Ludan, *et al.*, Interpretable-by-Design Text Understanding with Iteratively Generated Concept Bottleneck. *arXiv preprint arXiv:2310.19660* (2023).
17. OpenAI, GPT-4o System Card. Available at: https://openai.com/index/gpt-4o-system-card/ (2024).
18. Qwen Team, Qwen2.5-72B-Instruct. Available at: https://huggingface.co/Qwen/Qwen2.5-72B-Instruct (2024).
